# The two sub-genomes of the allotetraploid frog *Xenopus laevis* are evolving under similar selective pressure in extant populations

**DOI:** 10.1186/s12864-025-12036-4

**Published:** 2025-10-07

**Authors:** Dareen Almojil, Vinu Manikandan, Nizar Drou, John Measey, Stéphane Boissinot

**Affiliations:** 1 New York University Abu Dhabi, Saadiyat Island, Abu Dhabi, United Arab Emirates; 2 Center for Genomics and Systems Biology, New York University Abu Dhabi, Saadiyat Island, Abu Dhabi, United Arab Emirates; 3 Centre for Invasion Biology, Department of Botany and Zoology, Stellenbosch University, Stellenbosch, South Africa

**Keywords:** *Xenopus laevis*, Polyploidy, Selection, Population genetic, Demography, Genome evolution

## Abstract

The model species *Xenopus laevis* is an allotetraploid species, whose genome consists of two sub-genomes (the L and S sub-genomes) that were inherited from its parental species. Previous studies comparing the genome of *X. laevis* with other species of the genus revealed that the L sub-genome was more conserved than the S sub-genome suggesting it has been evolving under stronger purifying selection. However, it remains unclear if this difference reflects evolutionary processes that are still at play in extant populations. To answer this question, we conducted the first genome-wide survey of variation in this species by re-sequencing 44 individuals from its native South African range at ~ 10 × coverage. We generated a dataset of ~ 260M SNPs, which constitutes a valuable resource for the *Xenopus* community. We found that the South African populations of *X. laevis* are highly structured and differentiated, reflecting ancient divergence followed by more recent admixture at contact zones. We also determined that the landscapes of variation of the L and S sub-genomes do not show any significant differences suggesting that the two sub-genomes are responding to evolutionary forces in a similar manner. In particular we showed that purifying selection and positive selection are acting identically on the two sub-genomes, suggesting that the sub-genomes of *X. laevis* are evolving under similar selective pressure. Since 60% of the ancestral homeologous genes have been retained in *X. laevis*, this result suggests that the function of those genes is conserved on both sub-genomes or that a large number of genes has experienced neo- or sub-functionalization.

**Supplementary Information:**

The online version contains supplementary material available at 10.1186/s12864-025-12036-4.

## Introduction

The African clawed frog *Xenopus laevis* has long been used as a model system in developmental and cell biology [1, 2] but it is also an excellent model to study the evolutionary consequences of polyploidization in vertebrates. Polyploidization or whole genome duplication (WGD) has been associated with lineage diversification [3–5], evolutionary novelties [6, 7], adaptation (particularly in unstable environments) [7, 8] and invasiveness [9]. WGD can result from the duplication of the same genome (autopolyploidy) or from interspecific hybridization (allopolyploidy). Following WGD, some gene duplicates will lose function and become pseudogenes, since there is little selective pressure to maintain genetic redundancy, while others can gain new function (neo-functionalization) or become specialized (sub-functionalization) [10]. With time, the unnecessary genetic material will be lost or pseudogenized and the ancestral polyploid will evolve into a functional diploid, i.e. a genome where most genes are found as single copy, although a small fraction can be retained as duplicates.

In general, polyploidization is rare in vertebrates yet it has had a profound impact on the early evolution of this group [11–14]. Two rounds of WGD occurred in the ancestor of all vertebrate lineages around 500MY ago and have provided the genetic material necessary for the evolution of a large number of evolutionary novelties, such as the development of a complex nervous system [12], and the evolution of specialized sensory systems (e.g., vision and olfaction) [13, 14]. These early events of polyploidization have profoundly affected the evolution of vertebrates, yet they are ancient and to understand the evolutionary consequences of WGD, and in particular the transition from a tetraploid to a functionally diploid genome, studies of more recent polyploidization events are necessary. WGD in *Xenopus* provides such an opportunity. The genus *Xenopus* contains 29 species, 28 of which being polyploids [15–18]. The model species *X. laevis* and its close relative are allotetraploids. Allopolyploidy in *X. laevis* results from the hybridization between two diploid species, each contributing a sub-genome (referred as the L and S sub-genomes). It has been estimated that the two ancestral species diverged ~ 34MY ago and the event of hybridization occurred 17-18MY before present [19] (although older estimates between 25-65MY have been proposed using different methods and different calibration points) [20–23]. The genome of *X. laevis* has been sequenced and the parental origin of the two sub-genomes was inferred from their composition in transposable elements, which goes back to their two progenitors [19]. In terms of cell division, the genome functions as a diploid since chromosomes are disomic, so the two homoeologous sub-genomes do not recombine with each other [19, 24]. Genomic analyses have shown that the two sub-genomes have evolved asymmetrically. The S sub-genome shows more intra-chromosomal genomic rearrangements, shorter gene coding regions, more deletions, and fewer intact and functional genes compared to the L sub-genome [19], this asymmetry is also observed at the cytogenetic level [25, 26]. Comparisons between *X. laevis* and the diploid species *X. tropicalis* [19, 21, 22, 25–29] as well as comparisons between *X. laevis* and other tetraploid *Xenopus* [22–24, 30, 31] showed that the S sub-genome has evolved under more relaxed selection than the L sub-genome, resulting in shorter genes and a higher rate of pseudogenization on the S chromosomes. However, these results are based on comparisons between species that diverged a long time ago (~ 48-65MY for the *X. laevis*/*X.tropicalis* comparison and ~ 17-40MY for the comparisons between *X. laevis* and related tetraploid species) and are not as informative with regards to the effects of selection on the sub-genomes at a more recent time scale and in extant populations of frogs.

Here we present the first genome wide study of genetic variation in *Xenopus laevis* across its native range in South Africa where most of its diversity is found [32–36]. Using whole genome resequencing, we reconstruct the population structure and demography of *X. laevis* in South Africa. We then characterize the landscape of variation of the two sub-genomes in order to test if the two sub-genomes are differentially subject to mutation, migration, drift and selection in modern populations. Specifically, we aim to determine if the S sub-genome is harbouring signs of reduced selective pressure compared to the L sub-genome as suggested by interspecific comparisons, or if the L and S sub-genomes have reached an equilibrium and the tetraploid genome of *X. laevis* are evolving under similar pressure in extant populations.

## Materials and Methods

### Sampling

Tissue samples of 44 *Xenopus laevis* were collected across the South African distribution of the species to assess the genome-wide level of variation in this species (Supplementary table S1 for detailed samples information). Our sampling was approved by the Institutional Animal Care and Use Committee at New York University School of Medicine (IACUC Protocol 20–0003) and at Stellenbosch University (protocol number SU-ACUD14-00028) and was conducted under local permits provided by the South African Authority (Permit number: OP 7/2021). All animals were photographed then euthanized using a bath of 0.5% MS-222 (Tricaine methanesulfonate) [37]. Tissue samples (i.e., muscle, heart, liver, skin, and intestine) were dissected and stored in RNALater. The identification of *X. laevis* was validated based on the clear morphological differences between *X. laevis* and *X. gilli,* the only confirmed sympatric species with *X. laevis* in South Africa.

### DNA extraction, whole genome sequence alignment and Single Nucleotide Polymorphisms (SNP) calling

Genomic DNA was isolated from the muscle tissue of 44 individuals using the DNeasy blood and tissue kit (Qiagen, Valencia, CA) as per the manufacturer’s protocol. Extracted DNA was quantified using Qubit dsDNA HS Assay Kit (Thermo Fisher Scientific). Paired end libraries were generated with NEB library prep kit using 80 ng of DNA per sample and sequenced to 10 × coverage on Illumina NextSeq 550 flow cells by the sequencing core facility at New York University of Abu Dhabi.

Raw FASTQ sequenced reads where first assessed for quality using FastQC v0.11.5 [38]. The reads where then passed through Trimmomatic v0.36 [39] for quality trimming and adapter sequence removal, with the parameters (*ILLUMINACLIP: trimmomatic_adapter.fa:2:30:10 TRAILING:3 LEADING:3 SLIDINGWINDOW:4:15 MINLEN:36*) [39]. The surviving trimmed read pairs were then processed with Fastp [40] to remove poly-G tails and Novaseq/Nextseq specific artefacts. Following the quality trimming, the reads were assessed again using FastQC. After quality control assessment the reads were aligned to the *X. laevis* v9.2 genome assembly available through the Xenbase database (reference genome; https://www.xenbase.org/entry/static-xenbase/ftpDatafiles.jsp), which was used as our reference genome. The alignments were performed using BWA-MEM v0.7.15 [41]. The resulting SAM alignments were then converted to BAM format using SAMtools v1.3.1 [42]. The BAM alignments were then processed through PICARD tools v2.5.0. Briefly, the BAMs were coordinate sorted, alignment stats were gathered, read groups were added, duplicates (PCR and Optical) were marked, and the final post processed BAMs were indexed using SortSam, CollectMultipleMetrics, AddOrReplaceReadGroups, and BuildBamIndex respectively.

SNP detection was performed using GATK v3.5 HaplotypeCaller [43], to produce a per sample GVCF (with the parameters -ERC GVCF -stand_emit_conf 10 -stand_call_conf 30 –genotyping_mode DISCOVERY). The per sample GVCFs were combined and genotyped using GATK’s v3.5 CombineGVCFs and GenotypeGVCFs respectively, and the average coverage of the genomic data was 11x (Supplementary Figure S1). Filtering was performed in VCFtools v0.1.16 [44]. We performed two filtering steps, a more stringent one (with the following criteria: minimum depth of coverage = 5x, maximum average depth of coverage = 20x, no more than 20% missing data across all samples, a threshold of 5% minor allele frequency, a minimum genotype quality score of 30) and a more relaxed one to ensure we recover low frequency SNPs and singletons (minimum depth of coverage = 5x, maximum average depth of coverage = 20x, no more than 20% missing data across all samples, all minor alleles were included, a minimum genotype quality score of 30). The unfiltered dataset consisted of ~ 260 million SNPs. The stringent filtering resulted in a dataset of ~ 800,000 SNPs while the more relaxed filtering approach produced a dataset of 130 million SNPs. The data are available at Sequence Read Archive (SRA) of the NCBI database https://www.ncbi.nlm.nih.gov/bioproject/PRJNA1279774 under accession number (PRJNA1279774).

### Population differentiation and genetic diversity analyses

Genetic structure was assessed using three approaches. First, a Bayesian clustering approach implemented in the software STRUCTURE [45] was conducted on a subset of ~ 10,000 SNPs and a value of K that ranged from 1 to 6 (i.e., K is the number of genetic clusters or populations that STRUCTURE is asked to find). The analysis was set to run with a burn-in period of 100,000 steps followed by a Markov Chain Monte Carlo (MCMC) iteration of 100,000 steps. The length of the burn-in period was verified by ensuring that the Ln *P(D*) (i.e. the estimate of the posterior probability of the data for a given K) and the likelihood of the runs had stabilized. The adopted model was the correlated allele frequencies and admixture models. The admixture model allows individuals in the sample to be assigned to single cluster or jointly to two or more clusters if their combined genotypes indicate admixture. Ln *P(D*) was used as a model choice criterion to select for the best value of K [46]. In addition, the **Δ**K method was used to detect the uppermost level of population structure when several hierarchical levels exist. **Δ**K is an ad hoc method that calculates the second order rate of change of the likelihood function with respect to K [47]. To infer the number of populations (K) represented in the dataset, the mean of Ln *P(D*) for each K value and the highest **Δ**K were plotted using the software HARVEST [48]. Second, Principal Component Analysis (PCA) was used to identify population structure using the R package ‘SambaR’ [49]. Third, an unrooted maximum-likelihood phylogeny was created using RAxML v8 [50] under the GTR-GAMMA model of sequence evolution, and 1000 bootstraps to evaluate support for the phylogeny with the highest likelihood. The generated tree was plotted based on a subset of ~ 100,000 SNPs and edited with SplitsTree v0.4 [51].

Summary statistics of genetic diversity (the nucleotide diversity π, Tajima’s D), heterozygosity and relatedness were calculated for each of the populations identified. Divergence between populations (*F*_*st*_ and d_xy_) were also calculated. All population pairwise statistics were produced using the R package ‘SambaR’ [49]. The whole genome scans were calculated in windows of 100Kb for three parameters, π, *F*_*st*_ and d_xy_, using python v2.7.18 to run the “popgenWindows.py” script provided by the Github of Simon Martin (https://github.com/simonhmartin/genomics_general). Allele frequencies were calculated using VCFtools v0.1.16 [44] and for each population the percentage of fixed, private, and shared alleles were calculated using a customized python script (Alleles.py) available in the project’s github page (https://github.com/dareen22/Xenopus-laevis-WGS).

### Mitogenome assembly and time calibrated phylogeny

The mitogenome of 44 *X. laevis* was assembled using MITObim v1.9.1. [52]. Low quality reads and adaptors were filtered using Trimmomatic v0.39 [39] similar to the WGS data described earlier. Then, MITObim iterations were used to generate the final alignment files with the best consensus, which were then imported into Geneious [53] for assembly quality checking. To calculate the diversification timing of *X. laevis* in South Africa we constructed a time calibrated mitogenome tree using Bayesian inference (BI) analysis with BEAST v2.7.1 [54]. The analysis was parameterised under predefined partition of unlinked substitution models, where each partition and evolution model were set to the calibrated Yule model parameter. The Markov chain Monte Carlo (MCMC) chain length was set to 1,000,000 iterations with the first 25% iterations being discarded as burn-in period. Run quality was assessed using Tracer v.1.7 [55], and the best tree with Maximum Clade Credibility (MCC) was extracted using TreeAnnotator v2.7 [54]. Tree calibration was set on the divergence between the subgenera *Silurana* and *Xenopus* estimated based on molecular and fossil data to be around 36MY (27-51MY) [56]. This estimate was used under a log normal distribution prior, with a minimum age of 27 MYA, standard deviation of 1.2 MYA, and 95% CI from 27–51 MYA.

### Demography

Estimated changes in the effective population size (N_e_) over time in each population was reconstructed using SMC + + (i.e., Sequentially Markovian Coalescent, a probabilistic model used to infer past demographic events based on genomic sequence data) [57]. SMC + + uses Pairwise Sequentially Markov Coalescent inferred from spatial polymorphisms along the genome to calculate variation in effective population sizes. SMC + + has the advantage of using unphased genomes and thus of limiting biases in effective population size estimates that could be generated from phasing errors [57]. For this analysis we used the less stringent filtering parameters to include rare variants, and we used non-admixed individuals only. The run was conducted independently for each of the populations identified by STRUCTURE, excluding admixed individuals and the Niewoudtville population. The demographic analysis was scaled using a mutation rate of 2.05 × 10^–9^ per site per generation [19] and a generation time of one year [58] to translate coalescence times into years.

### Landscape of variation of the L and S sub-genomes

We investigated the landscape of variation of the L and S sub-genomes to determine if the two sub-genomes are subject to different evolutionary processes. In particular, we were interested in determining if the S sub-genome is evolving under more relaxed selective pressure than the L sub-genome, as suggested by interspecific comparisons [19–21, 25–31].

First, we determined if the overall pattern of variation differs between the L and the S sub-genomes in order to test the hypothesis that one of the sub-genomes (presumably the S sub-genome) evolves under a more relaxed selection regime than the other; in case of relaxed selection acting on the S sub-genome, we expect a smaller effect of linked selection on genetic variation and hence a higher diversity. In contrast, the sub-genome that is subject to stronger selection is expected to exhibit a lower nucleotide diversity (π) and an excess of rare variants resulting in Tajima’s D leaning towards negative values. We also examined if the sub-genomes were differentially subject to drift and migration and therefore contributed differently to population differentiation. To this end, we performed the STRUCTURE analysis and we calculated estimators of differentiation (*F*_*st*_ and d_xy_) for each sub-genome independently. Summary statistics (π, Tajima’s D, *F*_*st*_ and d_xy_) and STRUCTURE analyses were calculated for each of the sub-genomes independently using SambaR [49] and STRUCTURE [45], respectively.

Second, we determined if the effect of background selection affected differentially genes located on the L and S sub-genomes. Purifying selection results in a reduced level of genetic variation in the vicinity of protein coding genes (i.e., background selection) but this effect should be less pronounced in case of relaxed selection. We estimated the nucleotide diversity around protein-coding exons in non-admixed individuals from the south-western Cape population, because it is the largest in our sample (Supplementary Figure S2). The first and last exons for each protein coding gene were extracted from the *X. laevis* GFF sequence file. An independent file was created for each exon category: first exons (n = 33,866), last exons (*n* = 33,866), and single exons genes (*n* = 7,729). For each exon, 10Kb of sequence was extracted on each side and the nucleotide diversity was calculated using a customized Perl script (https://github.com/vinumanikandan/Nucleotide-diversity) that conducts loops in VCFtools v0.1.16 [44] to calculate π statistic per site, which then takes the average of the per site π statistic to get an overall “per-exon” nucleotide diversity. Nucleotide diversity for each of the exon’s type was compared between the two sub-genomes and statistical significance was determined using the z-test [59] function in R v.4.2 and validated by plotting the distribution of the p-values.

Third, we determined if the two sub-genomes differed in the abundance and frequency of variants with different effect. If one of the two sub-genomes evolves under relaxed selection, we expect that mutations that are predicted to be deleterious based on their impact on protein-coding genes (missense mutations) and should be observed at higher frequencies than if they are linked to the more selectively-constrained sub-genome. To this end, the SNP vcf file was annotated for functional effects using SnpEff v4.3 [60] with the following parameters (snpEff -Xmx90g eff, -lof, -noMotif, xenopus). Comparison of the SnpEff annotations and functional prediction between the L and S sub-genomes for the two genetic clusters with the largest sample sizes (south-western Cape and north South Africa clusters) was conducted using non-admixed individuals only. SnpEff annotates the variants and calculates the effects they produce on known genes (e.g., amino acid changes). The annotated SNPs were classified based on the categorization of the SnpEff program [60]: 1) Variant by impact: High = disruptive, Low = harmless, and Moderate = non-disruptive, 2) Variants by function class: Missense (nonsynonymous) mutation and Silent (synonymous) mutation. The frequency distribution of the high, moderate, and low effect SNPs was calculated and plotted.

Fourth, we determined if genes linked to the L and S sub-genomes are more likely to be subject to positive selection and thus contribute differently to adaptation. Loci that are involved in local adaptation are more likely to show a higher level of differentiation (measured by *F*_*st*_) than the genomic average. If one of the two sub-genomes contains more “adaptation genes”, it will also exhibit a larger number of highly differentiated genomic regions. We compared two populations found at different elevations in the northern South African population, a moderately high elevation population (Botveld’s pond, 1016 m) and two populations near sea level (St.Lucia and Hluhluwe, 25-60m). We collected 32 *X. laevis* from each place (Supplementary Table S2 and Figure S3). Samples from each elevation were pooled and each pool was sequenced at a 32 × coverage. The pools were sequenced as described above. For filtration, SAMtools (version 1.7) [42] was used to convert alignment BAM files for each pool into a mpileup file. Then, two Perl scripts provided by PoPoolation (*identify-genomic-indel-regions.pl* and *filter-pileup-by-gtf.pl*) [61] were used to remove indels. The filtration parameters were set as follows: –min-coverage 4 –max-coverage 50 –pool-size 32. We calculated *F*_*st*_ across the entire genome in windows of 100Kb and we determined if the number of highly differentiated windows (top 1% of the distribution) differ between the L and the S sub-genomes. Genetic differentiation (*F*_*st*_) between the two elevation was estimated using PoPoolation2 (v1.201) [62], following an established pipeline provided by Errbii et al. [63]. Alignment files from the two pools were merged into a single mpileup file using SAMtools (version 1.7) [42]. Then, the merged file was converted into a synchronized file, a specific file format that stores allele frequencies for all positions in a reference genome and for all the analysed populations [62]. *F*_*st*_ values were computed using (fst-sliding.pl) from PoPoolation2 (version 1.201) in 100 K of non-overlapping windows across all chromosomes. Lastly, potential adaptation genes were identified. To this end, we extracted all protein coding genes that were within 10Kb of highly differentiated SNPs (*F*_*st*_ ≥ 0.15 which corresponds to the top 1%).

## Results

We investigated genome-wide variation in *Xenopus laevis* across its native South African range. To this end we performed whole genome resequencing at a coverage of ~ 10 × on 44 samples from 29 localities. The resulting dataset consists of around 260 million SNPs (before filtering). From this, we produced a dataset with a stringent filtering (800,000 SNPs) and one with a more relaxed filtering to capture low frequency variants (130 million SNPs).

### Population differentiation and genetic diversity analysis

**Fig. 1:**
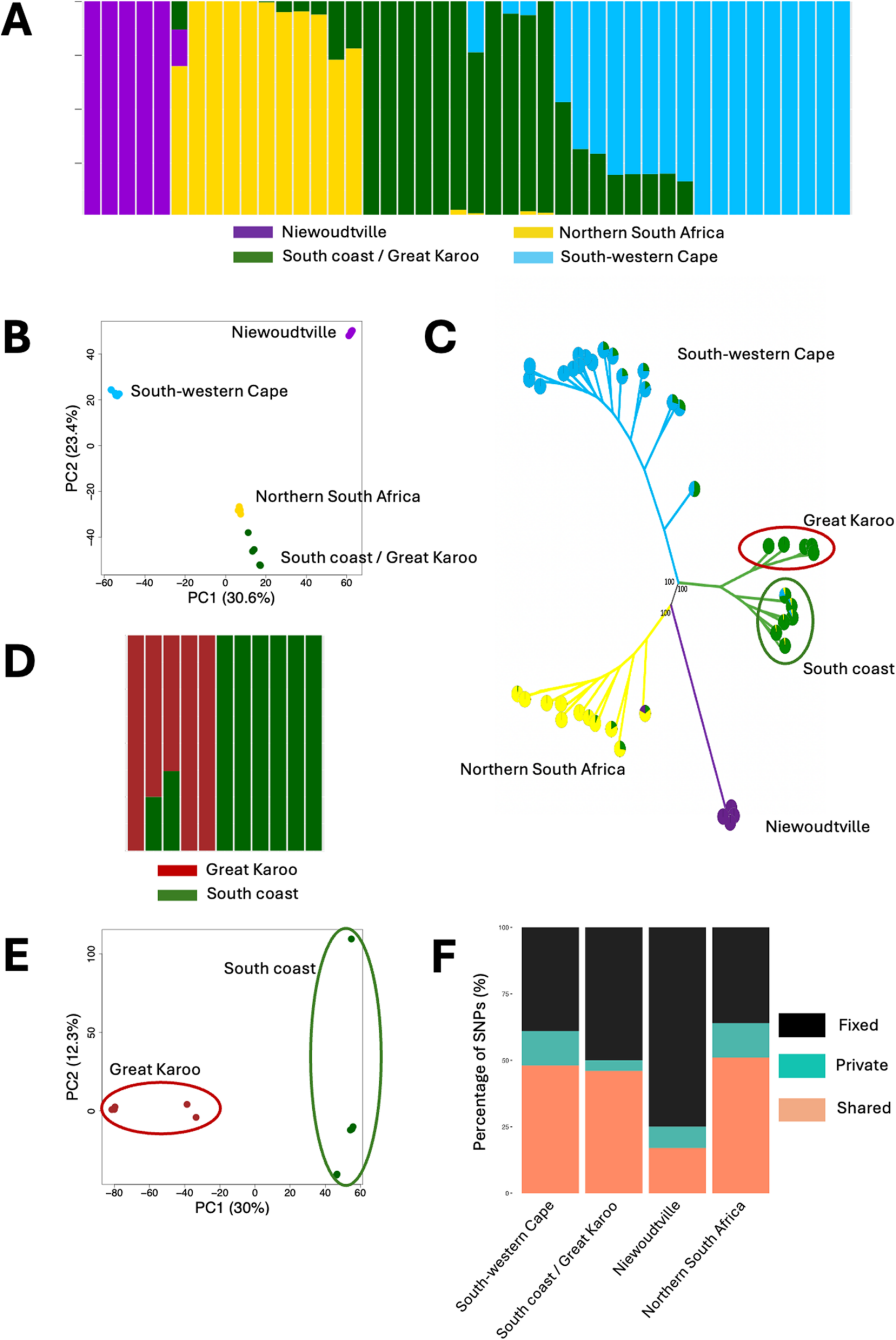
Population structure of *Xenopus laevis* in South Africa. **A**- STRUCTURE analysis showing 4 main clusters (K = 4). Each vertical bar represents one individual, and the proportion of colour within each bar indicates the individual’s estimated ancestry to each genetic cluster. The left ladder marks the proportion scale. **B**- PCA of SNP data showing the first two principal components (PC1 and PC2), which accounts for 30.6% and 23.4% of the total genetic variation respectively. Each point represents an individual, and clustering patterns reflect genetic similarity. **C**- Maximum likelihood phylogeny. **D**- STRUCTURE analysis showing that the Great Karoo and south coastal populations are genetically distinct. **E**- PCA of SNP data based on the Great Karoo and south coastal populations showing PC1 and PC2, which accounts for 30% and 12.3% of the total genetic variation respectively. **F**- Proportion of fixed, shared and private SNPs among populations

**Fig. 2:**
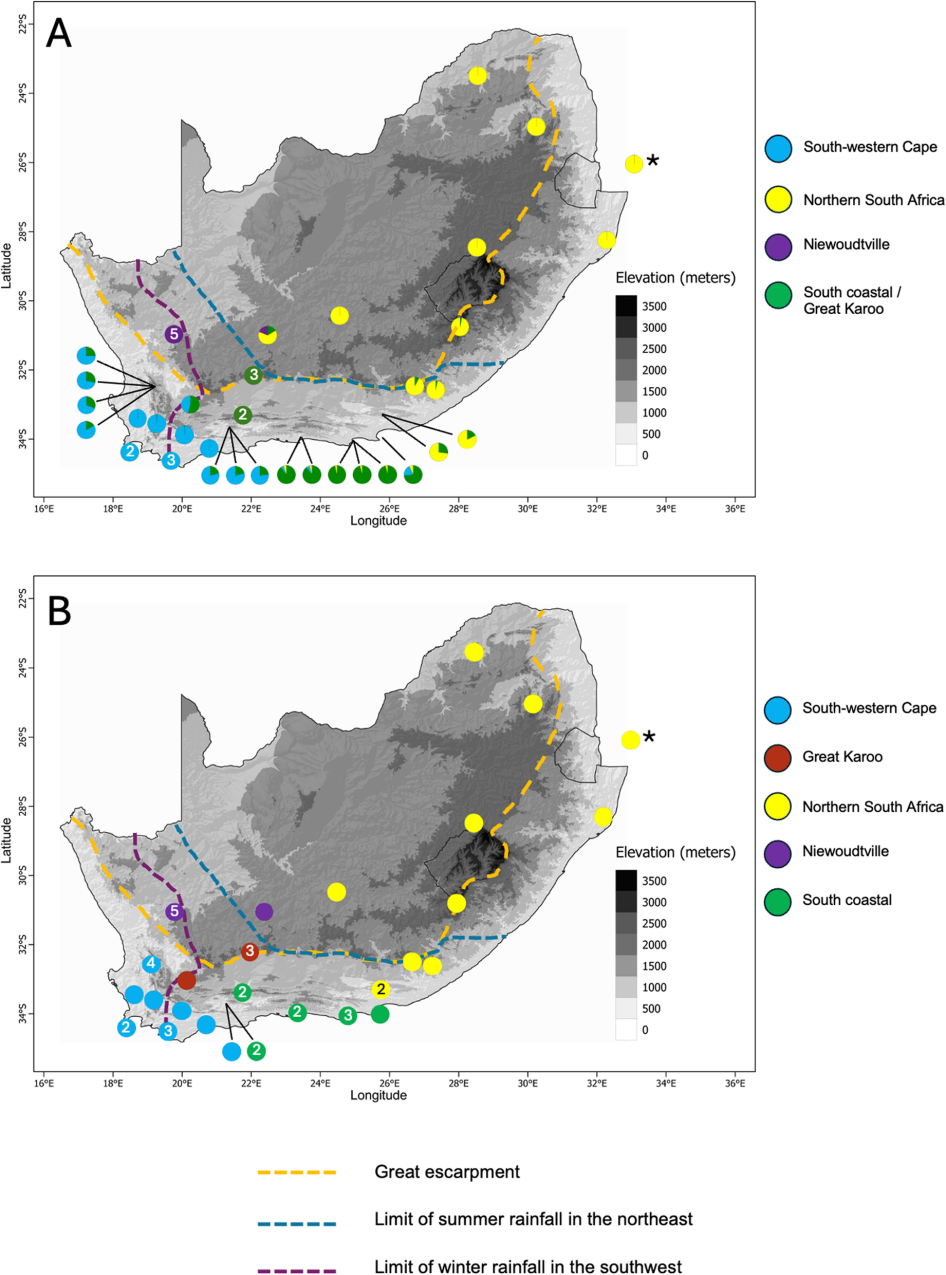
Geographic partitioning of genetic variation of *Xenopus laevis* in South Africa. **A**- Distribution of genetic variation based on nuclear SNPs. **B**- Distribution of mitochondrial clades. Numbers indicate the number of individuals from the same locality that harbour the same genetic composition

Using the stringent dataset of ~ 800,000 SNPs we first investigated the population structure of *X. laevis*. A STRUCTURE analyses (Fig. 1A; Supplementary Figure S4), a PCA (Fig. 1B) and a phylogenetic analysis (Fig. 1C) revealed the presence of four main genetic clusters with distinct geographic distributions (Fig. 2-A), which include (1) a south-western Cape population (in blue on Fig. 2-A) (2) a northern South Africa population (yellow) (3) a population that includes the south coast and extends to the great Karoo biodiversity region (green) and (4) a population near the town of Niewoudtville (purple). The STRUCTURE analysis reveals evidence of admixture at contact zones indicative of gene flow where the populations meet. Because the four main clusters correspond to highly differentiated entities (see below), we conducted additional STRUCTURE analyses on each of the main clusters, in order to detect more subtle genetic structure. This analysis revealed that the coastal and great Karoo populations are genetically distinct (Fig. 1-D). PCA analyses (Fig. 1-E), and phylogenetic analyses (Fig. 1-C) confirmed this result. The Niewoudtville population is represented by five individuals from the same pond with a very high level of relatedness and very low heterozygosity (Supplementary Figure S5). For this reason, this population was excluded from further analyses on population diversity and differentiation.

The populations show high level of differentiation (excluding admixed individuals) with values of *F*_*st*_ per 100Kb windows ranging from 0.14 between the south coastal and great Karoo populations to 0.39 between the southwestern Cape and the northern South Africa populations (Table 1). A large number of windows exhibited *F*_*st*_ values higher than 0.8, some of them even reaching 1 (Supplementary Figure S6), which is consistent with the large number of fixed SNPs between populations (Fig. 1-F). This high level of differentiation is also apparent in the values of d_xy_, which are ranging from 0.27 to 0.35 (Table 1). The genome-wide *F*_*st*_ and *d*_*xy*_ divergence have means that are similar to the medians (Supplementary Figures S6 and S7) which is a signal of homogeneous divergence across the whole genome.

**Table 1:** Summary statistics calculated for each *Xenopus laevis* populations. Admixed individuals were not included

Populations	Whole genome			L sub-genome			S sub-genome		
	_*π*_	*F*_*st*_	D_xy_	_*π*_	*F*_*st*_	D_xy_	*π﻿*	*F*_*st*_	D_xy_
SWC	0.14 ± 0.09			0.13 ± 0.07			0.13 ± 0.07		
NSA	0.20 ± 0.07			0.18 ± 0.06			0.18 ± 0.06		
SC	0.17 ± 0.07			0.17 ± 0.06			0.16 ± 0.07		
GK	0.16 ± 0.09			0.13 ± 0.07			0.12 ± 0.06		
SWC vs NSA		0.38 ± 0.19	0.34 ± 0.09		0.38 ± 0.18	0.30 ± 0.09		0.37 ± 0.18	0.34 ± 0.09
SWC vs SC		0.38 ± 0.18	0.32 ± 0.09		0.37 ± 0.17	0.32 ± 0.09		0.38 ± 0.18	0.32 ± 0.09
SWC vs GK		0.39 ± 0.18	0.35 ± 0.09		0.39 ± 0.18	0.33 ± 0.09		0.39 ± 0.18	0.34 ± 0.09
NSA vs SC		0.28 ± 0.16	0.30 ± 0.09		0.28 ± 0.16	0.30 ± 0.09		0.28 ± 0.16	0.30 ± 0.09
NSA vs GK		0.30 ± 0.18	0.35 ± 0.09		0.29 ± 0.16	0.29 ± 0.16		0.29 ± 0.16	0.31 ± 0.09
SC vs GK		0.14 ± 0.03	0.27 ± 0.07		0.14 ± 0.03	0.27 ± 0.07		0.14 ± 0.03	0.27 ± 0.07

*SWC* South western Cape, *NSA* Northern South Africa, *SC* South Coastal, *GK* Great Karoo, *π* Nucleotide diversity

**Fig. 3:**
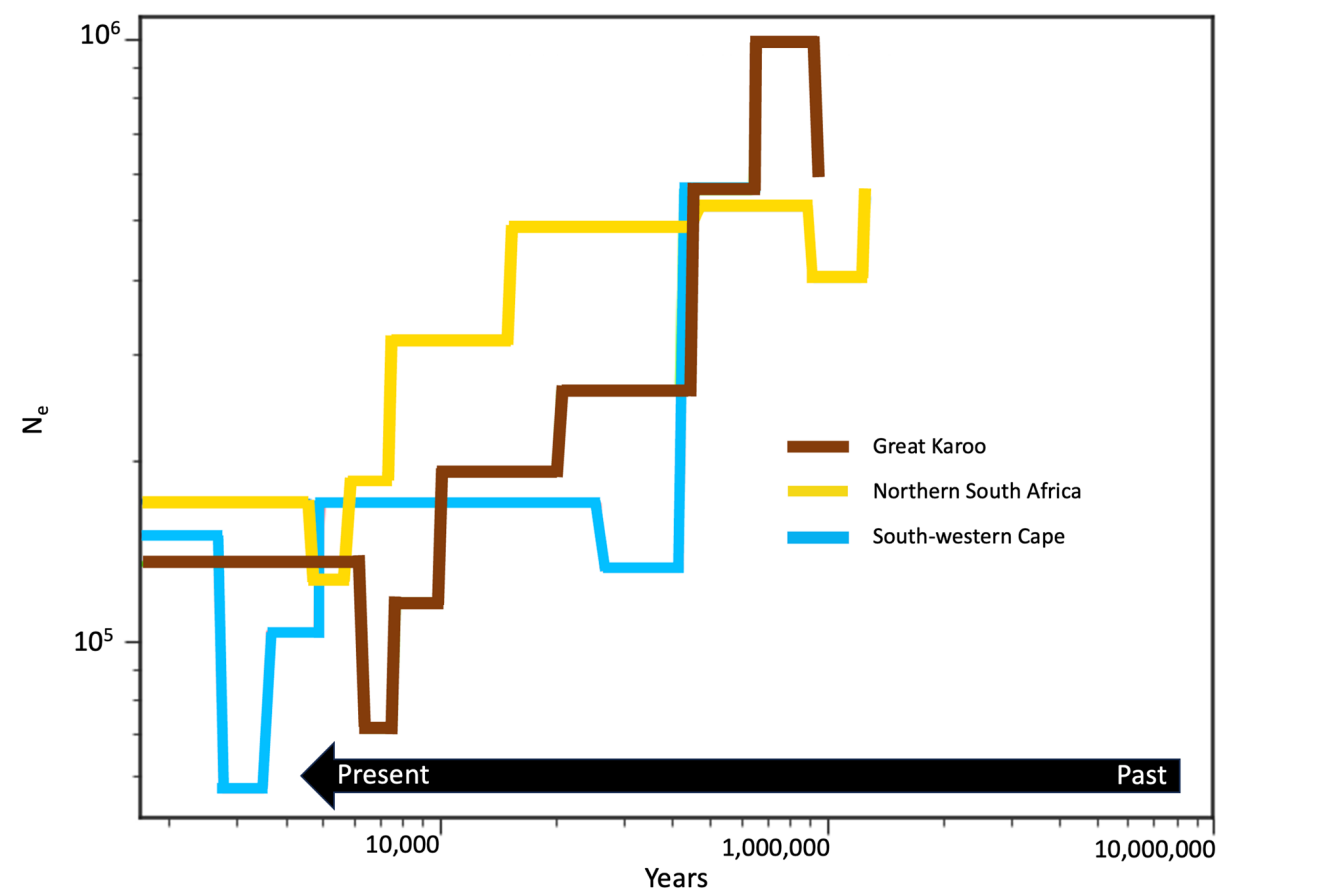
Demographic reconstruction of the three main population using SMC + + (Niewoudville population and admixed individuals were excluded). The right side of the figure represents past population size and the left side indicates more recent population size. N_e_ = effective population size

The nucleotide diversity differs among populations ranging from 0.14% in the south-western Cape population to 0.20% in the northern South Africa populations (Table 1; Supplementary Figure S8), however none of the differences in mean nucleotide diversity between populations are significant (Supplementary Figure S9). Values of Tajima’s D do not show any significant deviation from zero (Supplementary Table S3). A reconstruction of the demographic history of the three populations (excluding the Niewoudtville population and the south coastal population due to its small sample size and its admixed composition) using SMC + + (Fig. 3) reveals a general trend of population decrease over the last million year, with modern population size being roughly an order of magnitude lower than historical size. The decrease in the south-western Cape population seems to have occurred earlier than in the other two populations while the northern South Africa population has retained a larger population size longer than the other two populations, which is consistent with its higher nucleotide diversity.

**Fig. 4:**
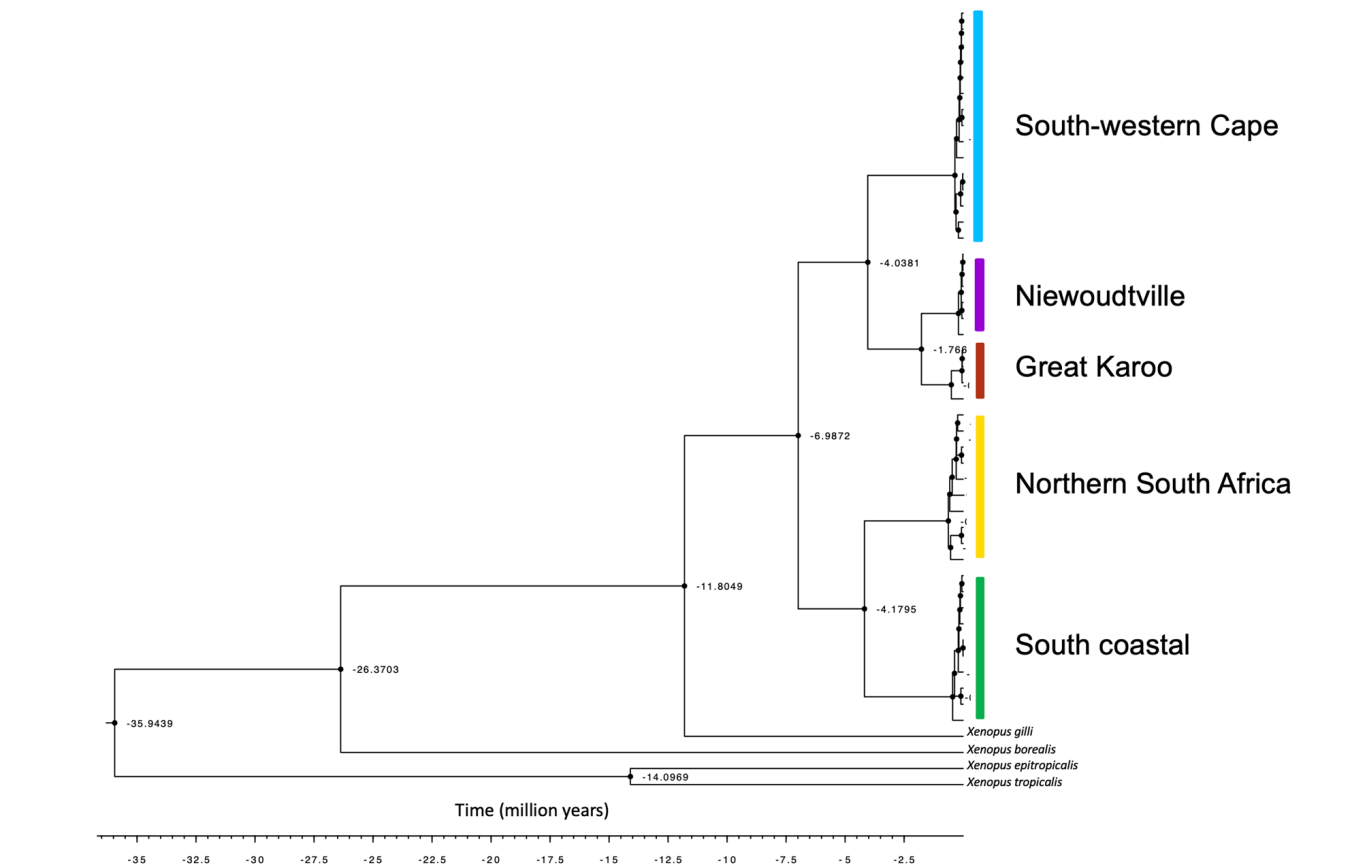
Dated mitochondrial phylogeny. The Calibrated Bayesian tree of mitogenome sequences was set on the divergence between *Silurana* and *Xenopus* estimated based on molecular and fossil data to be around 36Mya (27-51Mya) [56]

We also reconstructed the history of *X. laevis* using the complete mitochondrial genome. The mitochondrial phylogeny (Fig. 4) recovers five clades that diverged at different time (i.e., indicated by different colors in Fig. 4), whereby the geographic distribution coincides roughly with the genetic clusters identified using nuclear SNPs (Fig. 2-A and B). Using the *Silurana*/*Xenopus* as a calibration point, we estimated that the deepest split in the phylogeny of *X. laevis* in South Africa occurred ~ 7 MYA. This point divided the south western Cape, the great Karoo (from the West Beaufort location) and the Niewoudtville populations clade, from the northern South African population and the south coastal population, which also includes some individuals assigned to the great Karoo population in the nuclear analysis (from the Lainsburg and Matjelsfountain locations). It is interesting to note that the great Karoo and south coastal populations carry highly divergent mitogenomes while they were the least differentiated at the nuclear level. Interestingly, the second split occurred at ~ 4MYA, and simultaneously diverged the northern South Africa from the south coastal clade as well as the south western Cape from the great Karoo/Niewoudtville clades. The ancient divergence between populations estimated using the mitogenomes are consistent with the high level of differentiation observed at the nuclear level.

### Comparison of the landscape of variation of the L and S sub-genomes

We compared the pattern of variation and differentiation of the L and S sub-genomes to determine if they are subject to the same evolutionary forces. More specifically, our goal was to determine if the S sub-genome exhibits any signs of relaxed selection compared to the L sub-genome.

We first compared the nucleotide diversity (Table 1) and Tajima’s D (Supplementary Table S3) on each of the sub-genomes separately. The data on Table 1 shows how strikingly similar the values of nucleotide diversity are for all comparisons indicating that the landscape of variation does not differ significantly between the two sub-genomes. Similarly, values of *F*_*st*_ and d_xy_ are nearly identical for the L and S sub-genomes (Table 1; Supplementary Figures S6 and S7) and the STRUCTURE analysis showed that there is no difference in the genetic composition of each individual if the analysis is conducted on the L or on the S sub-genomes (Supplementary Figure S10). Together, these results indicate that the two sub-genomes seem to be equally affected by demographic (drift and migration) and selective processes, at least at this scale.

**Fig. 5:**
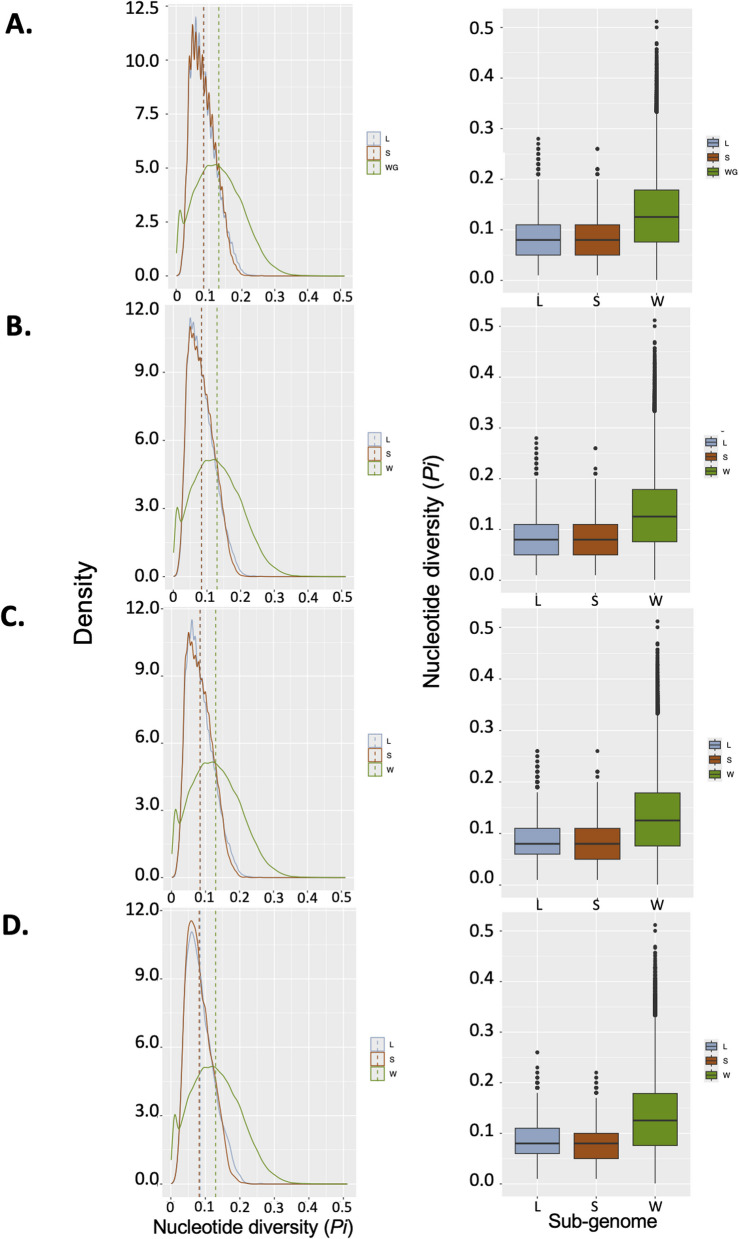
Comparison of nucleotide diversity between the L and S sub-genomes around exons. The left side of each panel shows the density distribution of nucleotide diversity for exons located on the L and S sub-genomes as well as for the whole genome average (W). The right side shows the same data as boxplots. **A**- All exons combined. **B**- First exons. **C**- Last exons. **D**- Single exon genes. The listed colors in the vignettes are light blue for the L sub-genome, brown for the S sub-genome, and green for whole genome data

To further examine possible differences in the effect of linked selection on the L and S sub-genomes, but at a smaller scale, we compared nucleotide diversity in the immediate vicinity of exons because this is where the effect of background selection is expected to be the more pronounced. To this end, we calculated the nucleotide diversity in 20Kb windows centered on the first and last exons of 33,866 multi-exons genes (19,762 on the L and 14,104 on the S sub-genome) and on 7,729 single-exon genes (4,464 on the L and 3,265 on the S). Figure 5 shows the comparisons in nucleotide diversity between the L sub-genome, the S sub-genome and the average for the entire genome. As expected, the nucleotide diversity is always lower near exons than the genomic average due to the effect of background selection. However, we didn’t detect any significant differences in nucleotide diversity between exons located on the L or S sub-genomes (Supplementary Figure S11). This analysis suggests that the landscape of variation of the L and the S sub-genomes is equally affected by background selection.

**Fig. 6:**
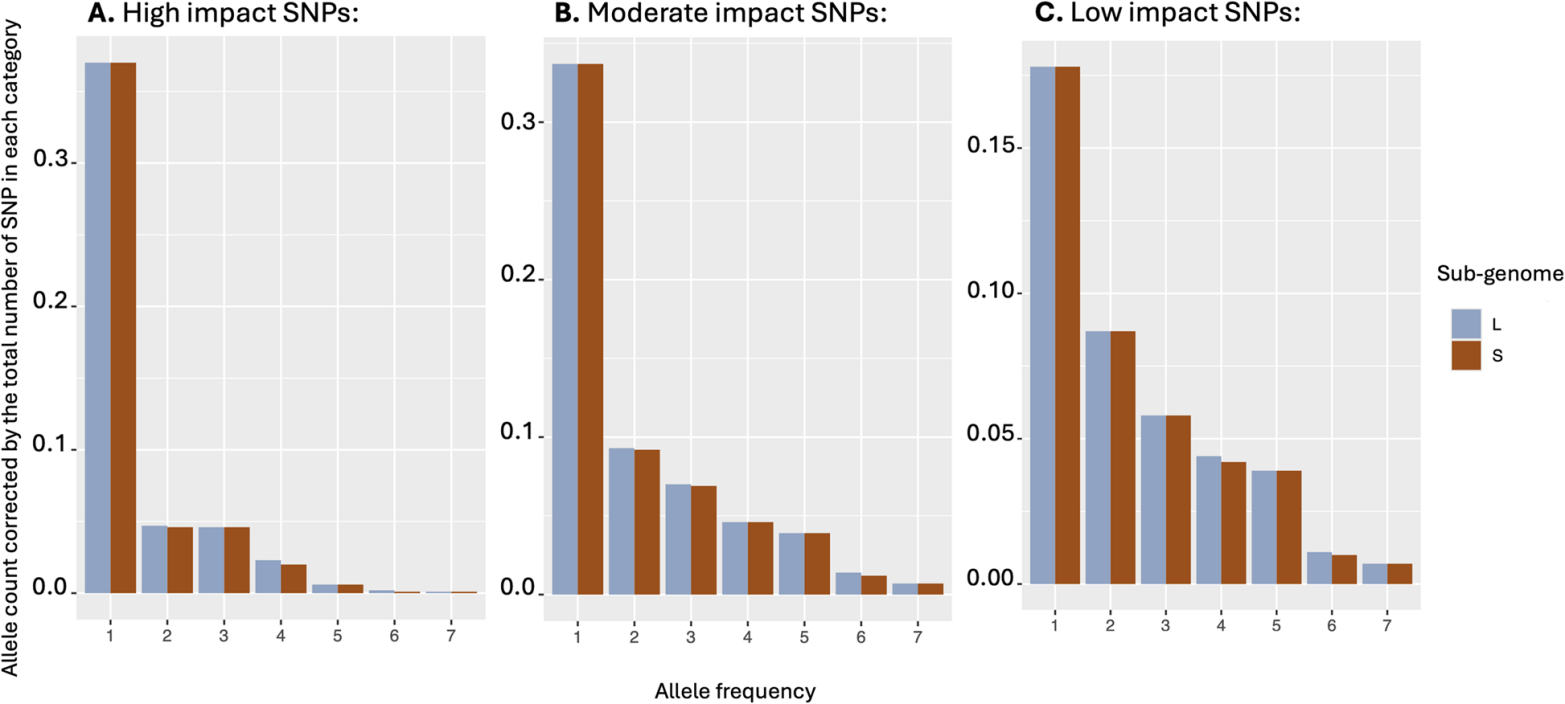
Allele frequency spectra for variants classified by SNPEff as high, moderate or low effects. The data are from the south western Cape populations and includes only non-admixed individuals

We also investigated the effect of selection directly at the protein-coding level. If the S sub-genome is subject to a more relaxed selective regime than the L, we should observe a higher fraction of mutations with a high or moderately high impact and these mutations should be found at higher frequency in the population than mutations linked to the L sub-genome. Similarly, we expect that missense (non-synonymous) mutations should be more common on the S than the L since they are more likely to be deleterious. We tested this hypothesis by classifying SNPs observed in the genome of non-admixed individuals from the south-western Cape and from the northern South Africa population. In both populations, there are no significant differences between the proportion of SNPs that have high, low and moderate impact between the two sub-genomes (Table 2). Similarly, there are no significant difference in the proportion of SNPs that are missense, or are silent and the ratio of missense/silent variation is nearly equal between the two sub-genomes (Table 2). We then investigated the frequency distribution of SNPs of different categories to determine if the most impactful SNPs are found at higher frequencies when carried by the S sub-genome than the L sub-genome. Figure 6 compares the frequency distribution of different types of mutations in the south-western Cape population due to its larger sample size. The frequency distribution is shifted to the left for the high impact SNPs relative to moderate and low impact SNPs, which is expected since these SNPs are more likely to be kept at low frequency due to the effect of purifying selection. Interestingly, the frequency distributions of SNPs of different effects are undistinguishable between the L and the S sub-genomes, indicating that selection is acting in a similar way on the different categories of SNPs.

**Table 2:** Comparison of the SnpEff annotations and functional prediction between the L and S sub-genomes for the south western Cape and northern South Africa populations (using non-admixed individuals only)

	South western Cape L sub-genome	South western Cape S sub-genome	Northern South Africa L sub-genome	Northern South Africa S sub-genome
Number of variants	13,071,518	11,304,094	43,613,473	37,260,032
Variants by Type				
SNP	10,433,646	9,043,623	35,005,219	29,948,424
Insertion	1,275,492	1,093,766	3,998,779	3,394,489
Deletion	1,362,380	1,166,705	4,609,475	3,917,119
Variants by Impact:				
High	21,169 (4.5%)	13,990 (4.2%)	58,253 (4.0%)	40,808 (4.0%)
Low	267,802 (56.7%)	191,259 (57.7%)	858,515 (59.3%)	607,369 (59.8%)
Moderate	183,702 (38.9%)	126,039 (38.0%)	529,973 (36.6%)	368,205 (36.2%)
Variant by function class:				
Nonsense	2,713 (0.7%)	1,864 (0.7%)	14,448 (1.2%)	9,915 (1.2%)
Missense	176,195 (46.4%)	120,092 (45.6%)	511,461 (43.7%)	353,447 (43.6%)
Silent	200,577 (52.9%)	141,421 (53.7%)	643,310 (55.02%)	446,736 (55.2%)
Missense/Silent ration	0.88	0.85	0.80	0.79

Finally, we investigated if the two sub-genomes are differentially subject to positive selection, and thus are differentially involved in adaptation. Genomic regions that are subject to different selective pressure should exhibit a higher level of genetic differentiation (measured by *F*_*st*_) than the genomic average. If one of the sub-genomes carries more regions that have been subject to positive selection, it should also exhibit a larger fraction of highly differentiated genomic regions. To test this possibility, we compared two populations from the northern South Africa cluster collected at moderately high elevation (~ 1000 asl) and around sea level and we calculated the mean *F*_*st*_ in non-overlapping windows of 100Kb, for a total of 232,644 windows. The mean *F*_*st*_ between the low elevation and the high elevation population is 0.09, with similar means for the S and L sub-genomes (0.09 for both). We selected the 1% most differentiated genomic regions between the two populations and determined the fraction that is L- or S-linked. We found approximately the same proportion of highly differentiated regions (2,506 windows) on the L and S sub-genomes (53% on the L vs 47% on the S) given the overall fraction of windows that were L- or S-linked (51% on the L sub-genome and 49% on the S sub-genome). Thus, it doesn’t seem that positive selection affects one sub-genome more than the other. There were however some differences between the sub-genomes, with a larger proportion of highly differentiated regions on chromosomes 3, 4, 5, and 8 of the L, but a higher proportion on chromosomes 2 and 7 of the S (Supplementary Figure S12). We also identified which genes were in close proximity (less than 10Kb) to the most highly differentiated SNPs (*F*_*st*_ > 0.15), which corresponds to the top 1% most differentiated SNPs. We identified 182 genes with functional annotation (Supplementary Table S4 and S5, for a list limited to the genes with *F*_*st*_ > 0.20). These include genes involved in functions related to high altitude adaptation such as skin pigmentation (*asip*), DNA repair in response to ionizing radiation (*rad51ap1*) and lipid metabolism (*apoa1*) but also to olfaction (*omp*) and immunity (*cd3g*). Among the 182 genes, 127 have retained copies on the L and the S sub-genomes, 42 are found only on the L sub-genome and 13 only on the S sub-genome, which corresponds roughly to the proportions expected considering that the S sub-genome has lost 31% of its genes and the L sub-genome 8% [19]. For the 127 genes that have homeologous copies on the L and S sub-genomes, we observe a strong signal of differentiation (and presumably directional selection) on both L- and S-linked copies for only four genes (*asip*, gpx3, or2w3 and *tshb*). For all other genes represented on the L and the S sub-genomes, the signature of selection was found on only one of the homeologs, suggesting that these copies are not functionally equivalent and possibly underwent sub-functionalization or neo-functionalization.

## Discussion

We conducted the first genome-wide survey of genetic variation in natural population of the African clawed frog *Xenopus laevis*. This resequencing effort yielded a collection of ~ 260 million SNPs, which constitutes a valuable source of information for the community of scientists using *X. laevis* as a model species. The analysis of SNP variation in *X. laevis* allowed us to investigate two questions: (1) the population structure, genetic diversity, demography and timing of diversification of the species in its native South African range and (2) the effect of selection on the L and S sub-genomes.

### A genome-wide perspective on Xenopus laevis evolutionary history

The genome-wide analysis of *X. laevis* across its native range in South Africa reveals a complex population structure shaped by climatic and geological events. Using a diverse of approaches, we identified four genetically distinct clusters with clear geographic distributions, with sub-structure between the Great Karoo and the South coastal areas. The identification of the four major genetic clusters is consistent with previous studies based on nuclear and mitochondrial markers [31–34] but our analysis provided higher resolution, revealing that the Great Karoo and the coastal populations are genetically distinct. This distinctiveness is consistent with morphological studies that showed that male *X. laevis* from the north of the Cape fold mountains are lighter, shorter and have less testis mass than males from the south region of the mountains [64]. We also found similar differences; the coastal population being bigger and heavier than the Karoo population (Supplementary Figure S13).

South African populations of *X. laevis* are highly differentiated, with high *F*_*st*_ values, particularly between the south-western Cape and northern South African populations (*F*_*st*_ = 0.39) and a large number of windows reaching 1. Consistent with these high *F*_*st*_ values, a large number of SNPs are fixed between populations. The homogeneous distribution of genome-wide *F*_*st*_ and d_xy_ values (Supplementary Figures S6 and S7) indicates that divergence is widespread across the genome rather than being restricted to specific regions. This means that genetic differentiation between populations is primarily shaped by historical isolation and demographic processes, rather than by local adaptation. These results suggest that populations have been evolving independently for extended periods of evolutionary time, with little to no gene flow, and it is only relatively recently that these populations came in contact, resulting in admixed populations at contact zones. A deep divergence time is supported by the mitochondrial dating which indicates that mitochondrial lineages could have split between four and seven million years, although the divergence between populations has to be more recent.

The presence of highly divergent mitochondrial haplotypes among the great Karoo and the coastal populations, despite their relatively low nuclear differentiation, suggests that these populations may have experienced historical isolation followed by secondary contact. This pattern of mito-nuclear discordance is consistent with a scenario in which previously isolated lineages have undergone partial nuclear admixture while retaining their distinct mitochondrial histories [65, 66]. Such patterns have been observed in the Coastal Reed Frog (*Hyperolius substriatus*) where hybridization and asymmetrical introgression influence mitochondrial and nuclear genomic variation differently [67].

The geographic distribution of the *X. laevis* populations presents similarities with other taxa (reviewed in [68]). In these taxa, populations or species have distributions matching the south-western Cape, northern South Africa and south coast subdivisions in *X. laevis*, suggesting that common biogeographic or climatic barriers have affected these species. In the case of *X. laevis*, it is likely that a combination of geological and climatic events has played a role in the diversification of the populations. The estimated divergence times between mitochondrial lineages provide valuable context for understanding the evolutionary history of *X. laevis* in South Africa. The deepest split at ~ 7 MYA suggests that the major lineages have been evolving independently since the late Miocene, which corresponds to a period of climatic and ecological instability in Africa, including increased aridification and habitat fragmentation [69–71]. In South Africa, dry periods could have caused the contraction of population ranges and thus made divergence in allopatry possible while more recent changes in precipitation could have favoured population expansion and secondary contacts. In addition, the increased aridity in South Africa that started in the Miocene has continued until present and has probably reduced the availability of favourable habitats for frogs that rely on waterbodies for their reproduction. This could explain the general decline in population size over the past million years we observed in the SMC + + analysis.

It has been proposed that the emergence of rainfall seasonality [72] may have contributed to the divergence between populations of *X. laevis* in South Africa, or at least to the persistence of distinct gene pools. This hypothesis is supported by the observation that the borders between the south western Cape, south coast and north South Africa populations roughly match the limits of the winter and summer rainfalls (Fig. 2) and by translocation experiments that demonstrate adaptation to different rainfall regimen [73]. Finally, it is likely that the complex geological history of South Africa has played an important role. In particular the Great Escarpment constitutes a major biogeographic barrier that could have limited gene flow between population and allopatric divergence between coastal populations (south-western Cape and south coast) and populations north of the Escarpment. Interestingly, the simultaneous divergence events at ~ 4 MYA, separating the south-western Cape clade from the great Karoo/Niewoudtville clade and the northern South Africa clade from the great Karoo coastal clade, may reflect responses to a tectonic geological uplift in the great escarpment that took place ~ 5 MYA [74–77] and created a barrier to migration.

### The L and S sub-genomes are subject to similar evolutionary forces

Our comparisons of genetic diversity of the L and S sub-genomes reveal that there are no differences among these sub-genomes in terms of differentiation and diversity, and this at multiple scales. The landscape of variation between the L and S sub-genomes is identical at the scale of the entire chromosomes and we demonstrated that the effect of background selection, the intensity of purifying selection against putatively deleterious alleles and the signature of positive selection doesn’t differ between the two sub-genomes. This indicates that the L and S sub-genomes are subject to the same evolutionary forces in extant populations of *X. laevis.* These results appear to contradict previous studies that showed an asymmetric evolution of the sub-genomes, the S sub-genome evolving under relaxed selection compared to the L-sub-genome. Inter-specific comparisons found that the S sub-genomes had shorter gene coding regions, more deletions, and fewer intact and functional genes compared to the L sub-genome, which is consistent with relaxed selection [19–21, 25–31]. However, an investigation of the effect of selection on L and S-linked genes revealed that differences in the selective regimen experienced by the L and the S sub-genomes differ through time and among lineages [31, 78]. Interestingly these studies found that the L and S sub-genomes experienced similar levels of purifying selection in the clade consisting of *X. laevis*, *X. largeni* and *X. allofraseri*, which is consistent with our observations. In contrast, purifying selection was acting more strongly on the L sub-genome in ancestral branches of the *Xenopus* phylogeny as well as in the *X. clivii*/*X. borealis* clade.

Together these results suggest that the two sub-genomes of *X. laevis* are subject to the same evolutionary forces and that the *X. laevis* sub-genomes are evolving under similar pressure. Following WGD, tetraploids lose genes since there is little selection to maintain genetic redundancy and, with time, they lose most of the redundant genetic material. This is exactly what is observed in yeast [79], vertebrates [10–13] and Brassicaceae [80], which have only retained 8 to 30% of the homeologous gene pairs, an exception being *Paramecium* which has kept 50% of homeologous genes [81, 82]. In the case of *X. laevis*, it is possible that the redundant genetic material that could have been lost without a fitness cost was lost soon after tetraploidization but that the remaining duplicates persist to maintain epistatic interactions among the protein products of those duplicates and are thus evolving under purifying selection [83, 84]. Another non-exclusive possibility is that there may have been an advantage in preserving the tetraploid status of most genes in a more recent evolutionary time, in particular if those genes underwent neo- or sub-functionalization. An indication this may be the case comes from our study of highly-differentiated genes, *i.e.* putative adaptation genes. We found that only 4 genes out of 127 exhibited a signature of directional selection on both L- and S-linked copies, while for the other 123 genes only one of the copies, the L or the S, showed a sign of high differentiation. It is however surprising that the majority of genes in the *X. laevis* genome would have evolved novel function or would have become specialized. A possible explanation is that the two populations we compared are very similar genetically (genome wide *F*_*st*_ = 0.09) and we may miss signal of selection on homeologous copies. Additional comparisons between populations that are more divergent will be necessary to confirm this result. More studies on gene expression and on the evolution of gene networks in *Xenopus* will also be necessary to determine the extent of neo- or sub-functionalization in the *Xenopus* genome.

## Conclusion

This study presents the first genome-wide survey of genetic variation in natural populations of *Xenopus laevis*, yielding over 260 million SNPs and providing a foundational genomic resource for this key model organism. Through comprehensive analyses, we uncovered a complex population structure shaped by South Africa’s climatic and geological history, revealing four genetically distinct lineages and evidence of deep divergence and secondary contact. Our results highlight how environmental and biogeographic barriers have driven long-term isolation and differentiation in *X. laevis*, consistent with patterns observed in other African taxa. Importantly, we found that the L and S sub-genomes of *X. laevis* experience equivalent evolutionary pressures, with no evidence of relaxed selection in the S sub-genome, contrasting with earlier interspecific studies. These findings suggest that *X. laevis* sub-genomes has stabilized over time and undergoing similar selection pressure. Collectively, this work advances our understanding of genome evolution following whole-genome duplication and contributes essential insights into the evolutionary history, population genomics, and functional architecture of an important amphibian model system.

## Supplementary Information

Supplementary Material 1. Table S1. List of samples used in this study. Table S2. Composition of the samples used in the pooled data analysis. Table S3. Values of Tajima’s D for X. laevis populations in South Africa. Table S4. List of genes in close proximity (<10Kb) to highly differentiated SNPs (Fst>0.15) between the high and low altitude pools. Table S5. List of the 47 identified genes in close proximity (<10Kb) to highly differentiated SNPs (Fst>0.2) between the high and low altitude pools. Figure S1. Whole genome re-sequencing quality control of the un-filtered data. Figure S2. Flow chart describing the comparison of nucleotide diversity between exons linked to the L and S sub-genomes. Figure S3. Pooled data sampling location in South Africa. Figure S4. Number of populations (K) inferred using the software STRUCTURE. Figure S5. Relatedness and heterozygosity among populations. Figure S6. Differentiation (Fst) of the L and S sub-genomes between the four populations. Figure S7. Genomic divergence (dxy) of the L and S sub-genomes between the four populations. Figure S8. Nucleotide diversity of the L and S sub-genomes for each of the four populations. Figure S9. Histograms of multiple testing results for nucleotide diversity differences between pairs of populations. Figure S10. Population structure of X. laevis in South Africa for the L and S sub-genomes. Figure S11. Multiple testing of nucleotide diversity differences between the L and S sub-genomes for A) the first exons of genes, B) the last exons, and C) single exon genes. Figure S12. Chromosomal distribution of genes linked (<10Kb) with highly differentiated SNPs (*Fst*>0.15) between *the high and low contrasts within the *northern South Africa population. Figure S13. Differences in the Snout to Ventral Length (SVL; cm) and mass (g) between samples collected from arid sites (Great Karoo population) and samples collected from coastal sites (South coastal population).

## Data Availability

The sequencing data generated in this study are publicly available in the NCBI Sequence Read Archive (SRA) repository at: [https://www.ncbi.nlm.nih.gov/bioproject/PRJNA1279774](https:/www.ncbi.nlm.nih.gov/bioproject/PRJNA1279774) with accession number (PRJNA1279774). All customised scripts are available in the project’s github page at: 1) https://github.com/dareen22/Xenopus-laevis-WGS 2) https://github.com/vinumanikandan/Nucleotide-diversity

## Abbreviations

WGD: Whole Genome Duplication
SNP: Single Nucleotide Polymorphism
IACUC: Institutional Animal Care and Use Committee
NYUAD: New York Uniersity Abu Dhabi
CGSB: Center for Genomics and Systems Biology
SRA: Sequence Read Archive
NCBI: National Center for Biotechnology Information
MCMC: Markov Chain Monte Carlo
BI: Bayesian Inference
MCC: Maximum Clade Credibility
PCA: Principal Component Analysis
dxy: An Absolute divergence measure of the average number of nucleotide differences per site between two populations
