# Supplementary material for: The two sub-genomes of the allotetraploid frog Xenopus laevis are evolving under similar selective pressure in extant populations
Source: BMC Genomics. 2025 Oct 7;26:887. doi: 10.1186/s12864-025-12036-4 (PMC12505570; doi:10.1186/s12864-025-12036-4)
Supplement: Supplementary file 1 — Supplementary Material 1. Table S1. List of samples used in this study. Table S2. Composition of the samples used in the pooled data analysis. Table S3. Values of Tajima’s D for X. laevis populations in South Africa. Table S4. List of genes in close proximity (<10Kb) to highly differentiated SNPs (Fst>0.15) between the high and low altitude pools. Table S5. List of the 47 identified genes in close proximity (<10Kb) to highly differentiated SNPs (Fst>0.2) between the high and low altitude pools. Figure S1. Whole genome re-sequencing quality control of the un-filtered data. Figure S2. Flow chart describing the comparison of nucleotide diversity between exons linked to the L and S sub-genomes. Figure S3. Pooled data sampling location in South Africa. Figure S4. Number of populations (K) inferred using the software STRUCTURE. Figure S5. Relatedness and heterozygosity among populations. Figure S6. Differentiation (Fst) of the L and S sub-genomes between the four populations. Figure S7. Genomic divergence (dxy) of the L and S sub-genomes between the four populations. Figure S8. Nucleotide diversity of the L and S sub-genomes for each of the four populations. Figure S9. Histograms of multiple testing results for nucleotide diversity differences between pairs of populations. Figure S10. Population structure of X. laevis in South Africa for the L and S sub-genomes. Figure S11. Multiple testing of nucleotide diversity differences between the L and S sub-genomes for A) the first exons of genes, B) the last exons, and C) single exon genes. Figure S12. Chromosomal distribution of genes linked (<10Kb) with highly differentiated SNPs (Fst>0.15) between the high and low contrasts within the northern South Africa population. Figure S13. Differences in the Snout to Ventral Length (SVL; cm) and mass (g) between samples collected from arid sites (Great Karoo population) and samples collected from coastal sites (South coastal population). [file 12864_2025_12036_MOESM1_ESM.zip › Almojil_etal_Supplementray _material.docx]

**Table S1.** **List of samples used in this study**. “Population” indicates the assignment based on the nuclear DNA; Individuals with an admixed ancestry based on the STRUCTURE analysis are indicated with “*”. “MtDNA” indicates the mitochondrial clade each individual belongs to (NSA=Northern South Africa clade; GK=Great Karoo; SC=South Coastal; SWC=South western Cape; NWV=Nieuwoudtville)

| **ID** | **Population** | **MtDNA** | **Lat.** | **Long.** | **Locality** | **Province** |
| --- | --- | --- | --- | --- | --- | --- |
| XL1 | Northern South Africa | NSA | -28.07 | 32.31 | Bonamanzi Lodge | KwaZulu Natal |
| XL2 | Northern South Africa* | NSA | -33.33 | 25.68 | Addo National Park | Eastern Cape |
| XL3 | Northern South Africa* | NSA | -30.80 | 27.96 | Rhodes | Eastern Cape |
| XL4 | Northern South Africa | NSA | -30.56 | 24.41 | South of Philipstown | Northern Cape |
| XL5 | Northern South Africa* | NSA | -32.67 | 26.48 | Fort Fordyce | Eastern Cape |
| XL6 | Northern South Africa | NSA | -25.47 | 30.12 | South of Dullstroom | Mpumalanga |
| XL7 | Northern South Africa* | NSA | -33.41 | 25.50 | Bontrug | Eastern Cape |
| XL33 | Northern South Africa | NSA | -26.01 | 32.95 | Inhaca Island | Mozambique |
| XL8 | Northern South Africa* | NSA | -32.85 | 27.23 | Dimbaza Dam | Eastern Cape |
| XL27 | Northern South Africa* | NWV | -31.35 | 22.30 | North of Loxton | Northern Cape |
| XL28 | Northern South Africa | NSA | -28.52 | 28.56 | Golden Gate Highlands | Free State |
| XL9 | Great Karoo* | GK | -32.41 | 21.96 | Beaufort West | Western Cape |
| XL10 | Great Karoo* | GK | -32.41 | 21.96 | Beaufort West | Western Cape |
| XL11 | Great Karoo | SC | -33.44 | 21.71 | North of Calitzdorp | Western Cape |
| XL12 | Great Karoo | SC | -33.44 | 21.71 | North of Calitzdorp | Western Cape |
| XL14 | Great Karoo | GK | -32.41 | 21.96 | Beaufort West | Western Cape |
| XL29 | South Coastal* | SC | -34.17 | 24.81 | St Francis Bay | Eastern Cape |
| XL30 | South Coastal* | SC | -34.14 | 24.81 | Kromrivier site | Eastern Cape |
| XL31 | South Coastal* | SC | -34.01 | 25.68 | Summerstrand | Eastern Cape |
| XL32 | South Coastal | SC | -34.14 | 24.81 | Kromrivier site | Eastern Cape |
| XL72 | South Coastal* | SC | -33.99 | 23.43 | Keurboomstrand | Western Cape |
| XL73 | South Coastal* | SC | -33.99 | 23.43 | Keurboomstrand | Western Cape |
| XL80 | South Western Cape* | GK | -33.13 | 20.34 | West of Matjiesfontein | Western Cape |
| XL13 | South Western Cape* | SWC | -32.56 | 19.38 | Matjiesrivier Nature Reserve | Western Cape |
| XL15 | South Western Cape* | SWC | -32.53 | 19.27 | Kromrivier Cederberg Park | Northern Cape |
| XL16 | South Western Cape | SWC | -34.39 | 20.84 | Witsand | Western Cape |
| XL17 | South Western Cape | SWC | -34.66 | 19.54 | Pearly Beach | Western Cape |
| XL18 | South Western Cape | SWC | -33.95 | 19.83 | McGregor | Western Cape |
| XL19 | South Western Cape | SWC | -33.66 | 19.27 | Rawsonville | Western Cape |
| XL20 | South Western Cape* | SC | -33.73 | 21.56 | Rooiberg Lodge | Western Cape |
| XL21 | South Western Cape* | SWC | -33.73 | 21.56 | Rooiberg Lodge | Western Cape |
| XL22 | South Western Cape | SWC | -34.30 | 18.44 | Cape of Good Hope | Western Cape |
| XL23 | South Western Cape | SWC | -34.30 | 18.44 | Cape of Good Hope | Western Cape |
| XL71 | South Western Cape* | SC | -33.73 | 21.56 | Rooiberg Lodge | Western Cape |
| XL74 | South Western Cape | SWC | -34.67 | 19.59 | Pearly Beach | Western Cape |
| XL75 | South Western Cape | SWC | -34.67 | 19.59 | Pearly Beach | Western Cape |
| XL76 | South Western Cape* | SWC | -32.56 | 19.38 | Matjiesrivier Nature Reserve | Western Cape |
| XL81 | South Western Cape | SWC | -33.57 | 18.55 | East Saxonsea | Western Cape |
| XL77 | South Western Cape* | SWC | -32.53 | 19.27 | Kromrivier Cederberg Park | Northern Cape |
| XL78 | Nieuwoudtville | NWV | -31.33 | 19.89 | North of Calvinia | Northern Cape |
| XL79 | Nieuwoudtville | NWV | -31.33 | 19.89 | North of Calvinia | Northern Cape |
| XL24 | Nieuwoudtville | NWV | -31.33 | 19.89 | North of Calvinia | Northern Cape |
| XL25 | Nieuwoudtville | NWV | -31.33 | 19.89 | North of Calvinia | Northern Cape |
| XL26 | Nieuwoudtville | NWV | -31.33 | 19.89 | North of Calvinia | Northern Cape |

**Table S2.** **Composition of the samples used in the pooled data analysis.** All samples were collected from the North South Africa population. Each pool was sequenced at 32x coverage and is composed of 32 individuals. See supplementary figure S3 for location of samples on a map.

| **N** | **Sex** | **Group** | **Altitude (m)** | **Latitude** | **Longitude** | **Site** |
| --- | --- | --- | --- | --- | --- | --- |
| 2 | F | Low altitude | 25 | -28.34878 | 32.42925 | St Lucia Horse |
| 7 | M | Low altitude | 25 | -28.34878 | 32.42925 | St Lucia Horse |
| 15 | F | Low altitude | 60 | -28.03475 | 32.271356 | Hluhluwe sewage works |
| 10 | M | Low altitude | 60 | -28.03475 | 32.271356 | Hluhluwe sewage works |
| 15 | F | High altitude | 1016 | -29.37006 | 30.689734 | Botveld's pond, Dalton |
| 12 | M | High altitude | 1016 | -29.37006 | 30.689734 | Botveld's pond, Dalton |
| 5 | Juv. | High altitude | 1016 | -29.37006 | 30.689734 | Botveld's pond, Dalton |

**Table S3. Values of Tajima’s D for *X. laevis* populations in South Africa**. None of the values are significantly different from 0.

|  | Whole Genome | L sub-genome | S sub-genome |
| --- | --- | --- | --- |
| South-western Cape | -0.0139 | -0.0057 | -0.0084 |
| Great Karoo/South Coastal | -0.0185 | -0.0024 | -0.0073 |
| Northern South Africa | -0.0283 | -0.0064 | -0.0071 |
| Nieuwoudtville | -0.0634 | -0.0756 | -0.0715 |

**Table S5. List of the 47 identified genes in close proximity (<10Kb) to highly differentiated SNPs (*F_st_* > 0.2) between the high and low altitude pools.** “Chr” indicates on which chromosome the highly differentiated gene is located and “Copy” indicates if the gene is found on both sub-genomes or only one of them.

| **Gene** | **Chr.** | **Copy** | **Function** |
| --- | --- | --- | --- |
| CBWD1 | 1L | L and S | Enable GTPase binding activity and zinc chaperone activity. |
| GATC | 1L | Only L | Involved in the formation of correctly charged Gln-tRNA (Gln) to catalyze the transfer of glutamine. |
| TRIAP1 | 1L | L and S | Involved in the modulation of the mitochondrial apoptotic pathway by ensuring the accumulation of cardiolipin (CL) in mitochondrial membranes. Also, involved in mediating cell survival by inhibiting activation of caspase-9 which prevents induction of apoptosis. |
| APOA1 | 7S | L and S | Encodes apolipoprotein A-I, which is the major protein component of high-density lipoprotein (HDL) in plasma. |
| YIF1B | 8L | L and S | Involved in the regulating traffic from the endoplasmic reticulum to the plasma membrane and the organization of the Golgi architecture. |
| RAD51AP1 | 8L | Only L | Involved in DNA repair, cellular response to ionizing radiation, and positive regulation of DNA recombination. |
| SURF6 | 8L | Only L | Uncharacterized conserved protein. |
| PRSS2 | 7L | L and S | Encodes anionic trypsinogen. |
| CD3G | 7S | L and S | T-cell-specific surface glycoprotein precursor. |
| RPS3 | 2S | L and S | Plays a role in repair of damaged DNA. Also involved in the regulation of transcription, translation of mRNA, spindle formation and chromosome movement during mitosis, and apoptosis. |
| FOXD4L1 | 1L | L and S | Maintains an undifferentiated neural ectoderm after neural induction. |
| LSM4 | 1L | L and S | Small nuclear ribonucleoprotein (snRNP), also act as a co-factor in cell volume regulation. |
| SERPINH1 | 2S | L and S | Plays a role in collagen biosynthesis as a collagen-specific molecular chaperone. |
| IL10RA | 7L | L and S | Mediate the immunosuppressive signal of interleukin 10 and thus inhibits the synthesis of proinflammatory cytokines. |
| R3HDM4 | 1S | L and S | Enable nucleic acid binding activity. |
| NRM | 8L | Only L | Predicted to play a role in the suppression of apoptosis. |
| VPS28 | 2L | L and S | Regulates triglyceride synthesis via ubiquitination. |
| FAM206A | 6S | Only S | Predicted to be involved in dendrite morphogenesis, regulation of actin filament polymerization, and regulation of filopodium assembly. |
| COX6A1 | 1L | L and S | Cytochrome c oxidase (COX), the terminal enzyme of the mitochondrial respiratory chain; it catalyzes the electron transfer from reduced cytochrome c to oxygen. |
| PHLDA1 | 3L | L and S | Play a role in the anti-apoptotic effects of insulin-like growth factor-1. |
| HNRNPA3 | 9-10L | L and S | Predicted to be involved in mRNA splicing, via spliceosome. |
| SPAM1 | 3L | Only L | Involved in sperm adhesion. |
| EIF5A | 5S | L and S | Translation factor that promotes translation elongation and termination |
| OR1L4 | 3L | Only L | Involved in the initiation of a neuronal response that triggers the perception of a smell. |
| BTG4 | 7S | L and S | anti-proliferation factor that can induce G1 arrest in the cell cycle. |
| TSHB | 2S | L and S | Provides instructions for making the TSHB (Thyroid Stimulating Hormone = Beta Subunit) which is crucial for the control of thyroid structure and metabolism. |
| OMP | 2S | L and S | Associated with the mature olfactory receptor neurons which dictate the ability to respond to odour stimuli. |
| GSC2 | 1S | Only S | Predicted to be associated with development defect characterized by conotruncal heart defects and craniofacial anomalies. |
| ACER3 | 2S | L and S | Enables N-acylsphingosine amidohydrolase activity; calcium ion binding activity; and zinc ion binding activity. Involved in several processes, including myelination; positive regulation of cell population proliferation; and sphingolipid metabolic process. Mostly hydrolyzes sphingolipid ceramide to sphingosine and free fatty acid. |
| ABO | 8L | L and S | Encodes glycosyltransferase (ABO blood group system transferase) which catalyzes the transfer of carbohydrates to the H antigen. |
| CHST2 | 5L | L and S | Encodes a sulfotransferase protein and may play a role in biosynthesis of 6-sulfosialyl Lewis X antigen which is involved in immunity. |
| COLCA2 | 7S | L and S | Predicted to be involved in positive regulation of DNA-templated transcription. |
| GCLM | 4L | Only L | Involved in the synthesis of glutamate-cysteine ligase required for the synthesis of glutathione, which is involved in tissue building, repair and immune functions. |
| ACY3 | 2L | Only L | Predicted to enable aminoacylase activity and play a role in deacetylating mercapturic acids in Kidney proximal tubules. |
| NFE2L2 | 9-10L | L and S | Encoded transcription factor (bZIP: basic-leucine zipper) which regulates genes that play role in activating pathways to produce antioxidant in response to injury and inflammation, which includes the production of free radicals to combat oxidative stress. |
| STYXL1 | 2L | Only L | Involved in negative regulation of stress granule assembly, positive regulation of intrinsic apoptotic signalling pathway, and positive regulation of neuron projection development. |
| EEF2 | 1S | L and S | Involved in improving dense connective tissue repair and healing by regulating autophagy and apoptosis. |
| MYH8 | 8L | L and S | Encodes a member of the class II or conventional myosin heavy chains, and functions in skeletal muscle contraction. |
| MDH2 | 2L | L and S | Encodes Krebs cycle enzyme mitochondrial malate dehydrogenase, which catalyzes the reversible oxidation of malate to oxaloacetate, utilizing the NAD/NADH cofactor system in the citric acid cycle. Play a pivotal role in the malate-aspartate shuttle that operates in the metabolic coordination between cytosol and mitochondria. |
| ZNF593 | 2L | L and S | Enables preribosome binding activity and zinc ion binding activity. |
| GPX3 | 3S | L and S | Involved in catalyzing the reduction of organic hydroperoxides and hydrogen peroxide (H2O2) by glutathione, and thereby protect cells against oxidative damage. |
| ASIP | 9S | L and S | Agouti gene encodes a paracrine signalling molecule that causes melanocytes to synthesize pheomelanin, a yellow pigment, instead of the black or brown pigment, eumelanin. |
| PLA2G1B | 1L | L and S | Encodes a secreted member of the phospholipase A2 (PLA2) class of enzymes, which is produced by the pancreatic acinar cells. The enzyme may be involved in several physiological processes including cell contraction, cell proliferation and pathological response. |
| DAPK3 | 1S | L and S | Death-associated protein kinase 3 (DAPK3) induces morphological changes in apoptosis. |
| FAM110D | 2L | L and S | Enables alpha-tubulin binding activity. Involved in positive regulation of cell migration, positive regulation of phosphatidylinositol 3-kinase/protein kinase B signal transduction. and regulation of cell projection assembly. |
| PDIK1L | 2L | L and S | Predicted to enable serine/threonine kinase which plays a role in autophagy. Also predicted to be involved in chromatin remodelling and protein phosphorylation. |
| SLC30A2 | 2L | L and S | this gene is a zinc transporter that acts as a homodimer. |

Mean: 11.4

Median: 11.2

Mean: 12.5

Median: 12.1

Mean: 10.8

Median: 10.7

Mean: 10.9

Median: 10.5

Mean: 11.2

Median: 11.0

Mean: 12.4

Median: 12.0

Mean: 10.6

Median: 10.5

Mean: 10.7

Median: 10.3

All samples

South western Cape

Southern coast

Northern South Africa

L sub-genome

S sub-genome

F

E

C

B

D

A

SWC

GK/SC

NWV

NSA

**Figure S1. Whole genome re-sequencing quality control of the un-filtered data.** A) Variant quality - a measure of how much confidence we have in our variant calls based on Phred score (minimum threshold set for 30 Phred score). B) Variant missingness plotted as proportion of missing data per site. C) Boxplot showing the proportion of missing data per individual across populations (NSA=Northern South Africa; GK/SC=Great Karoo and South Coastal; SWC=South western Cape; NWV=Nieuwoudtville). D) Variant mean depth (i.e. the number of reads that have mapped to this position). E) Mean depth per individual calculated as the mean depth of coverage per individual. F) Comparison of the coverage depth of the two sub-genomes.


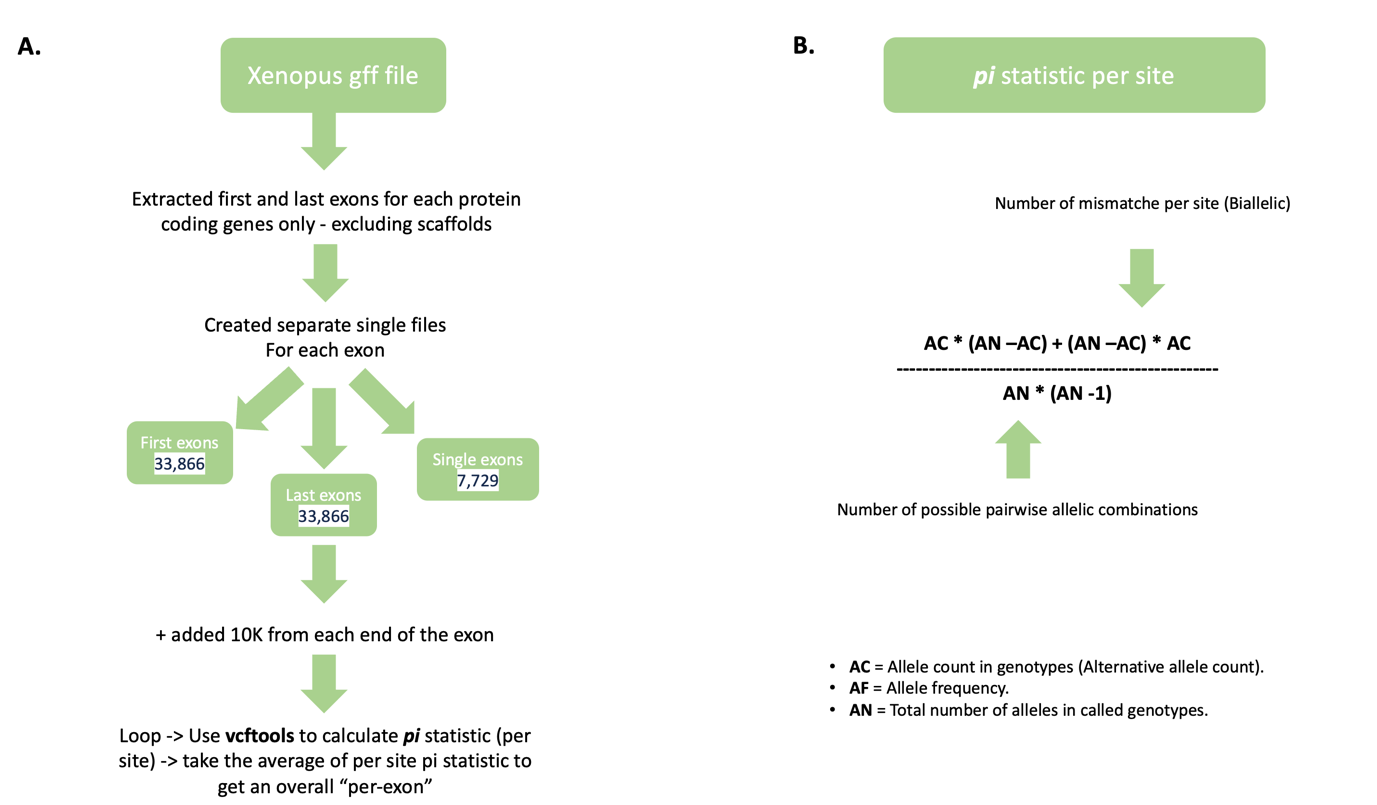


**Figure S2. Flow chart describing the comparison of nucleotide diversity between exons linked to the L and S sub-genomes.**


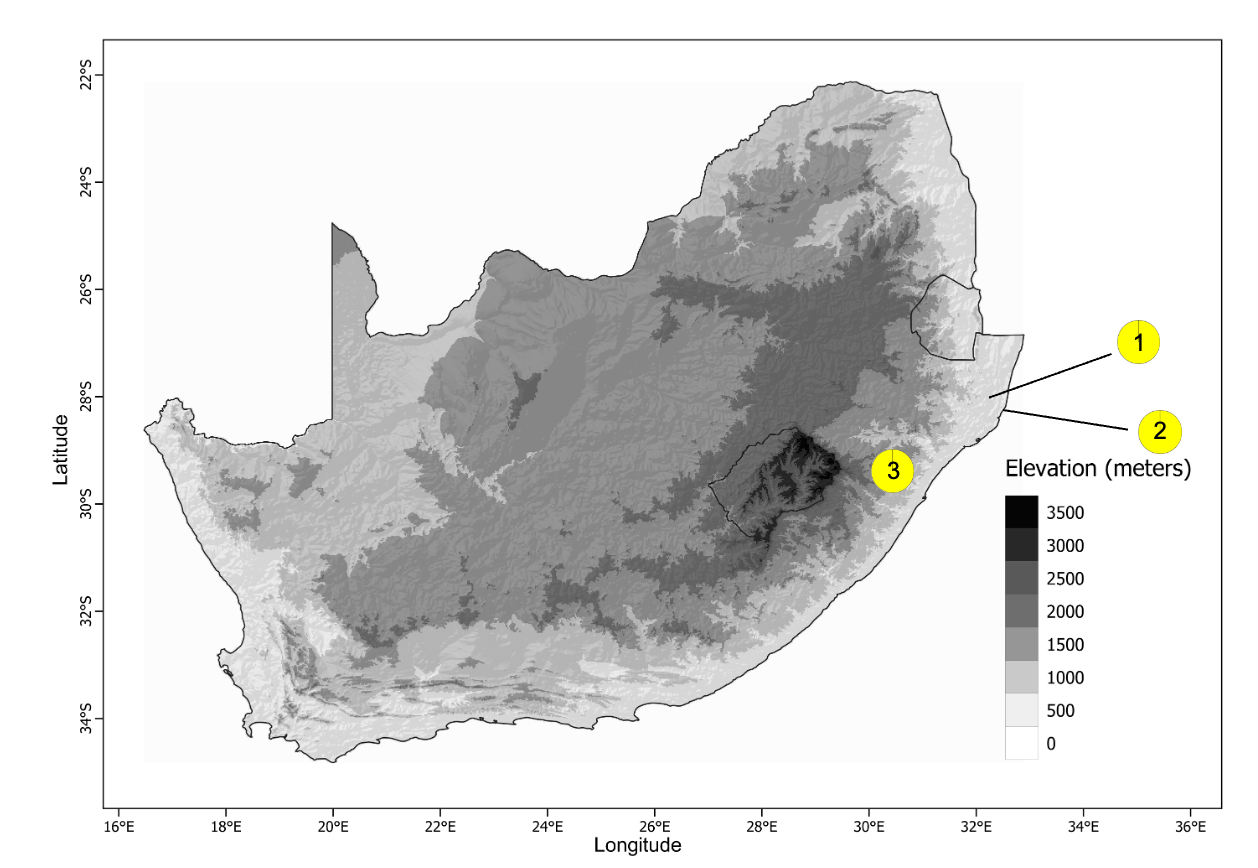


**Figure S3. Pooled data sampling location in South Africa.** 1= St. Lucia Horse (25m elevation); 2= Hluhluwe sewage (60m); 3= Botveld’s pond (1,016m). Details about the samples are available on Table S2.


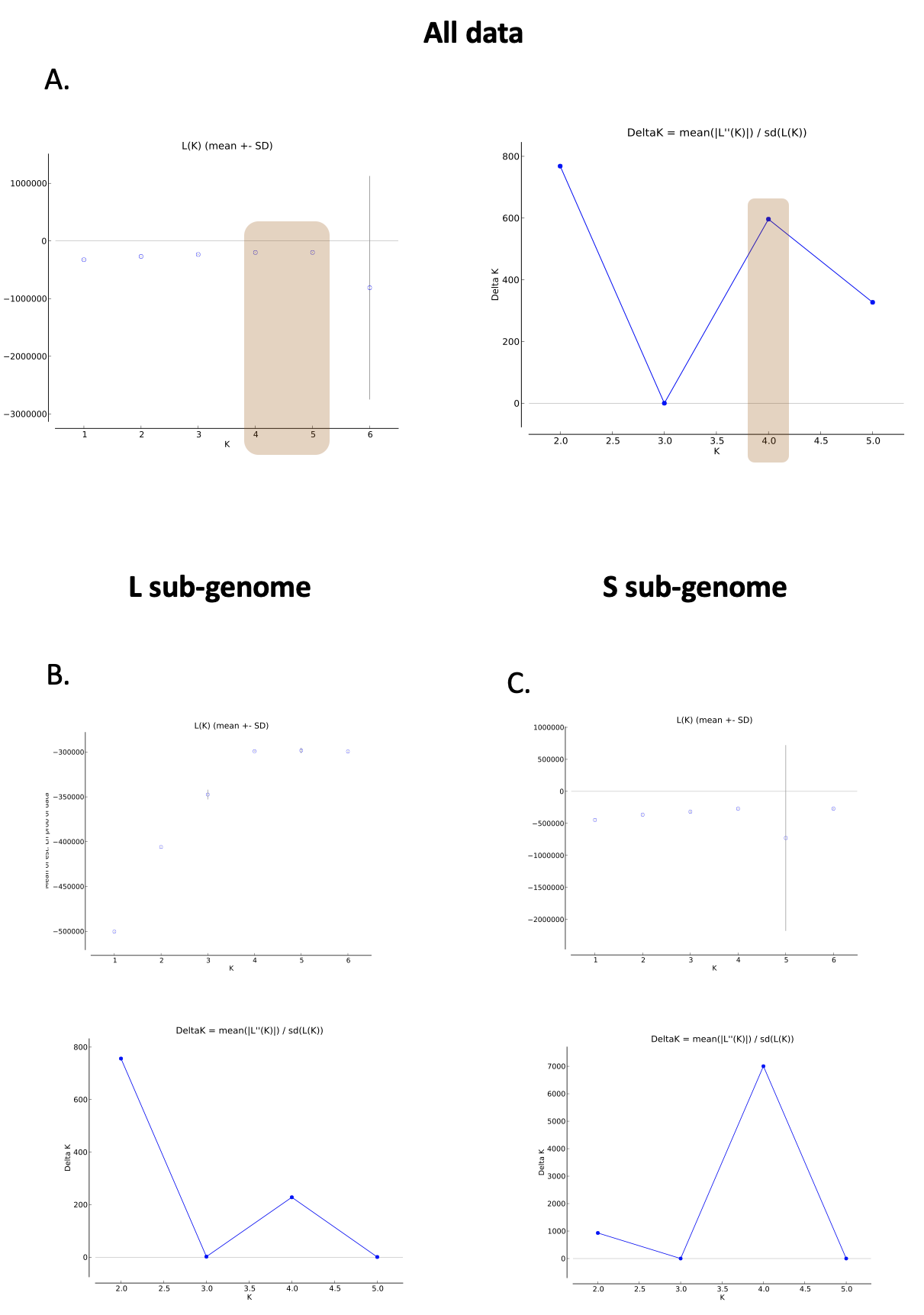


**Figure S4. Number of populations (K) inferred using the software STRUCTURE.** A) Inference based on the entire genome. On the left, a plot of the estimated mean probability value Ln *P(D*) for each *K* value plotted using the R statistical package 2.7.2 (R Development Core Team, Vienna, Austria). The Ln *P(D*) is used as a model choice criterion to select for the true value of K, the population inference is estimated at K=4 where a plateau in the value of Ln *P(D*) is observed. The figure on the right shows a plot of the highest **Δ**K which detect the upper most level of population structure when several hierarchical levels exist. ΔK is an ad hoc method that calculates the second order rate of change of the likelihood function with respect to K. B) Inference derived from using only the L sub-genome. C) Inference derived from using only the S sub-genome. (Parameters used: ancestry model = Admixture Model. Frequency Model = Allele frequencies are correlated among populations. Number of K’s: K = 1-6, number of iterations per K = 20).

**
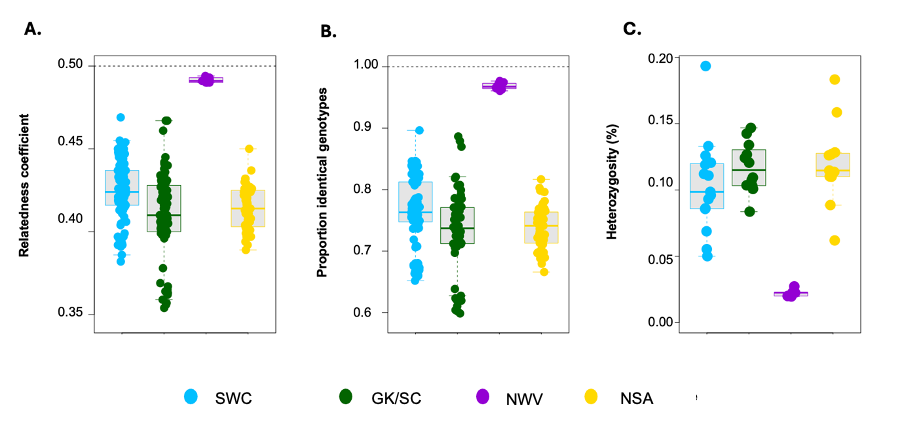
**

**Figure S5. Relatedness and heterozygosity among populations.** A) Relatedness coefficient measured among individuals of each population. B) Proportion of identical genotypes measured between two samples from SNP genotypes. Relatedness calculations are based on the proportions of genotype scores (0, 1, 2) of pairs of individuals. For biallelic SNPs, it is calculated based on three possible genotypes: (0: homozygous major allele, 1: heterozygous, 2: homozygous minor allele). C) Genome-wide heterozygosity measurements described as the percentage of heterozygous sites observed for each individual within the SNP dataset. (NSA=Northern South Africa; GK/SC=Great Karoo and South Coastal; SWC=South western Cape; NWV=Nieuwoudtville).


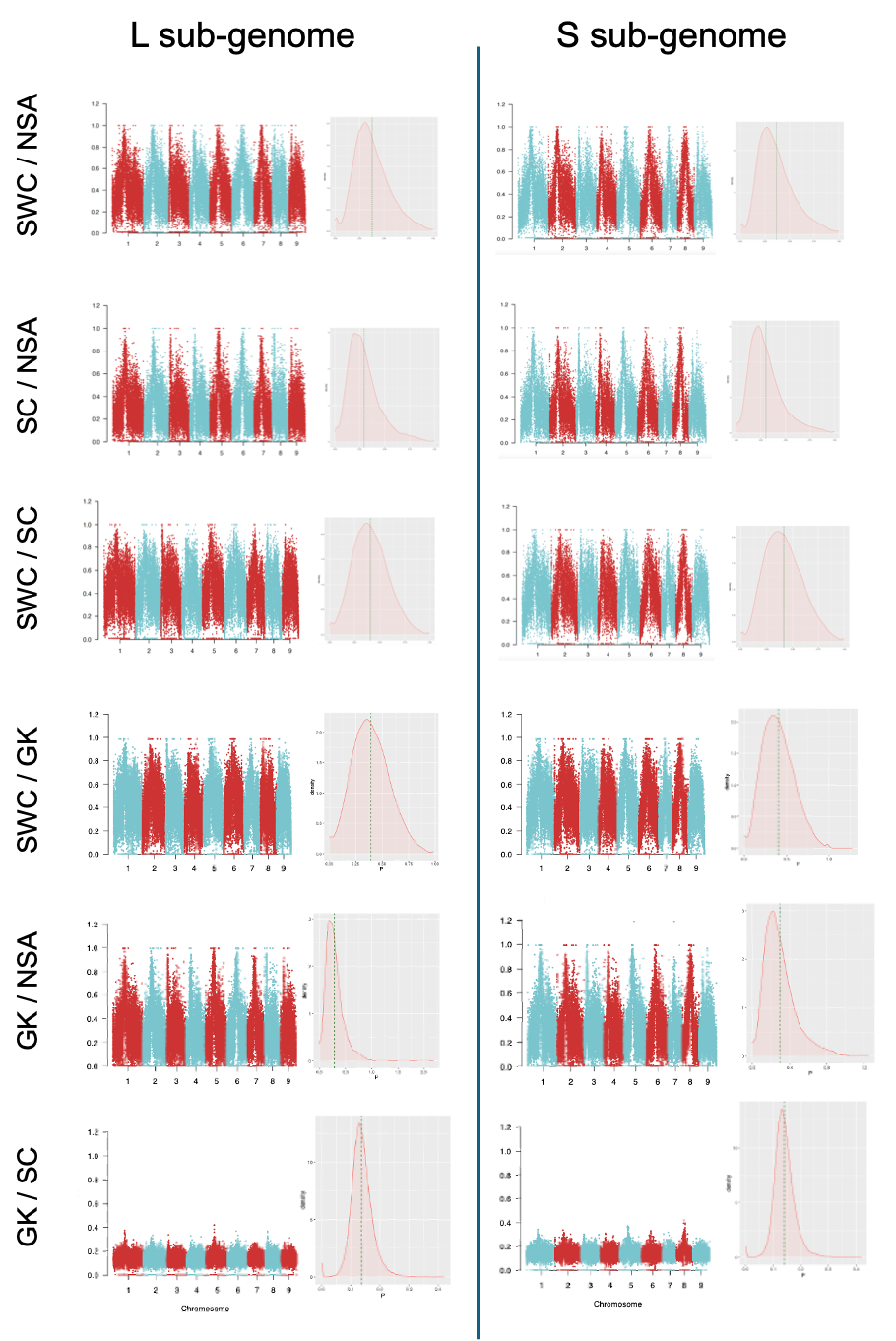


**Figure S6. Differentiation (*F_st_*) of the L and S sub-genomes between the four populations.** The *F_st_* values were calculated using the following parameters: Window size = 100 kb, step size = 25 k, minimum number of SNPs per window = 10 SNPs. For each pair of population and each sub-genome, the *F_st_* distribution along the chromosomes and the density distribution of *F_st_* are shown. NSA=Northern South Africa; GK/ =Great Karoo; SWC=South western Cape; SC=South coastal.


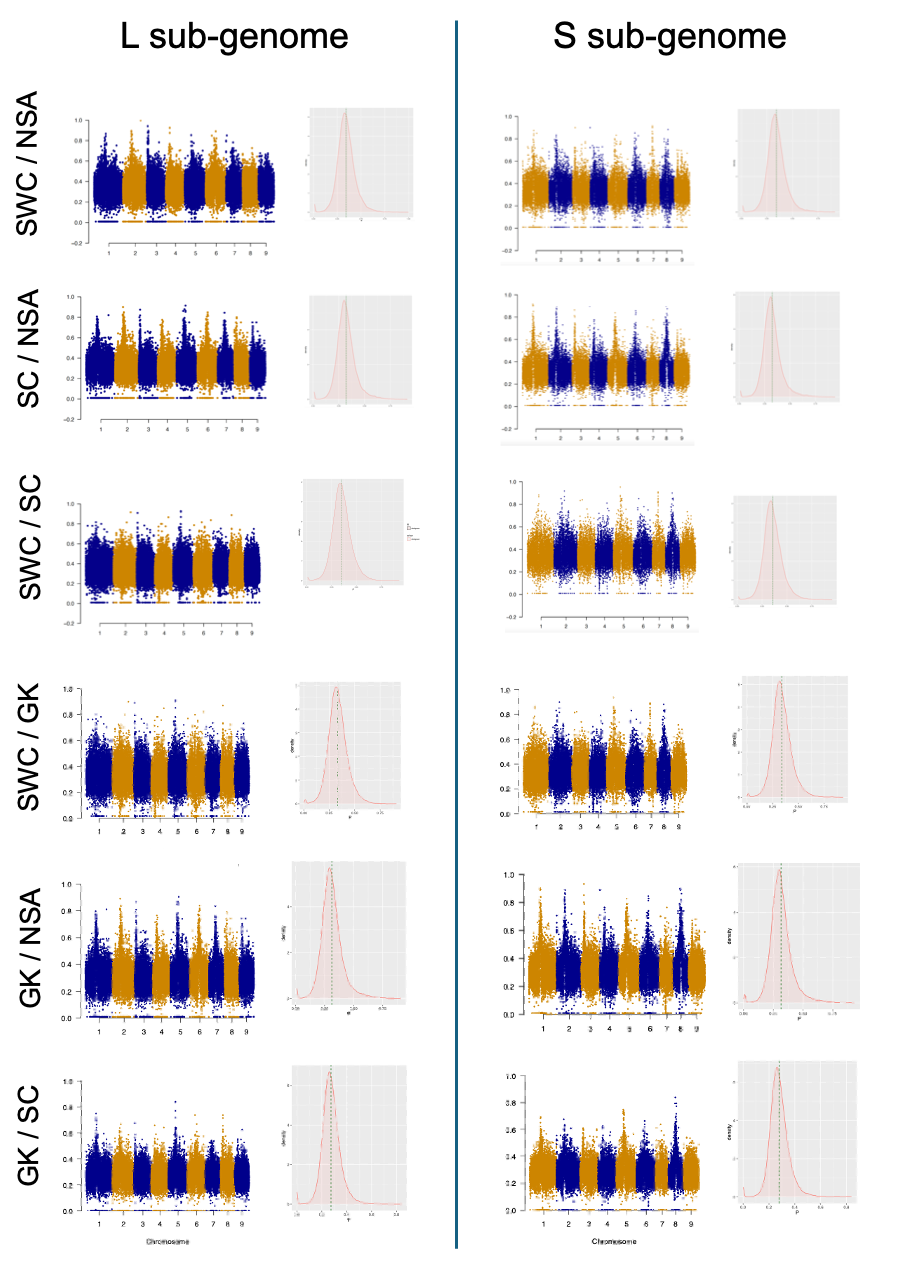


**Figure S7. Genomic divergence (dxy) of the L and S sub-genomes between the four populations.** The dxy values were calculated using the following parameters: Window size = 100 kb, step size = 25 k, minimum number of SNPs per window = 10 SNPs. For each pair of population and each sub-genome, the dxy distribution along the chromosomes and the density distribution of dxy are shown. NSA=Northern South Africa; GK/ =Great Karoo; SWC=South western Cape; SC=South coastal.


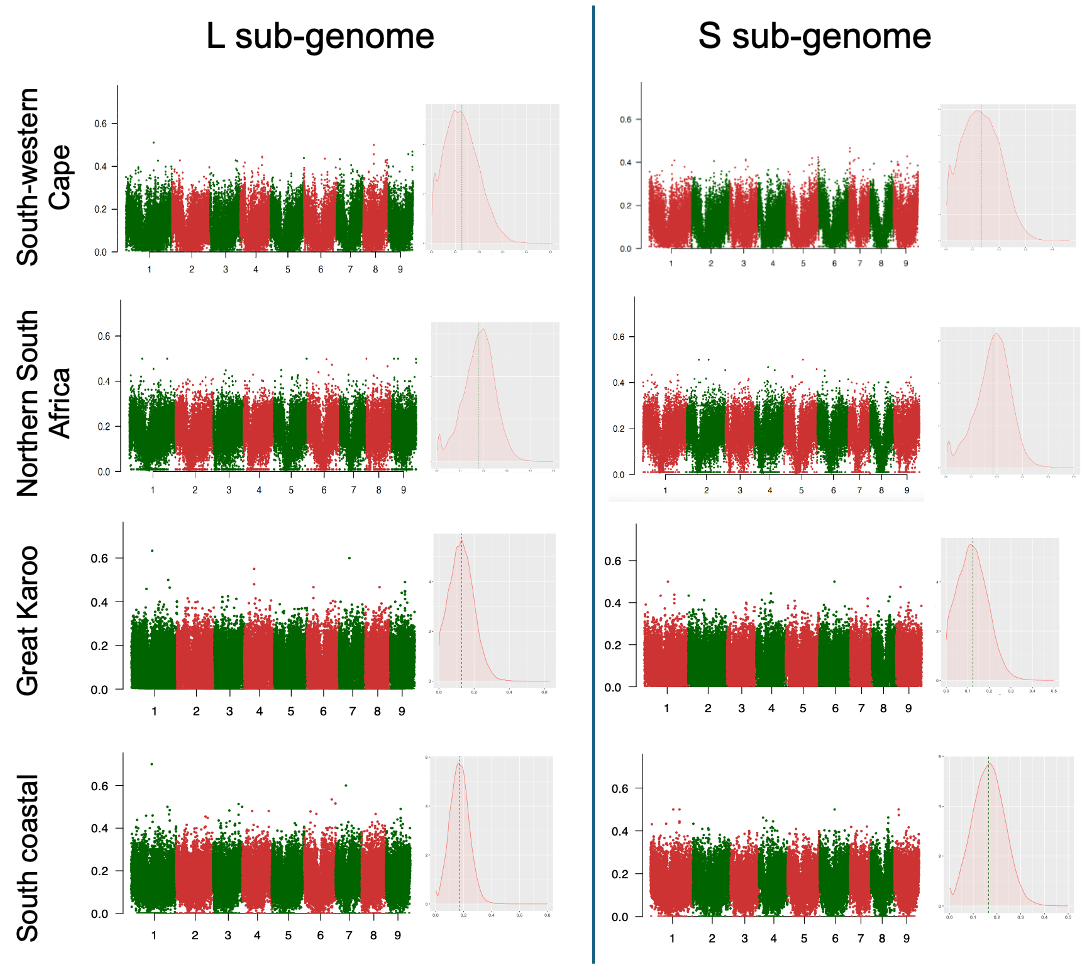


**Figure S8. Nucleotide diversity of the L and S sub-genomes for each of the four populations.** The values of nucleotide diversity were calculated using the following parameters: Window size = 100 kb, step size = 25 k, minimum number of SNPs per window = 10 SNPs. For each population and each sub-genome, the distribution of nucleotide diversity along the chromosomes and the density distribution of the diversity are shown.


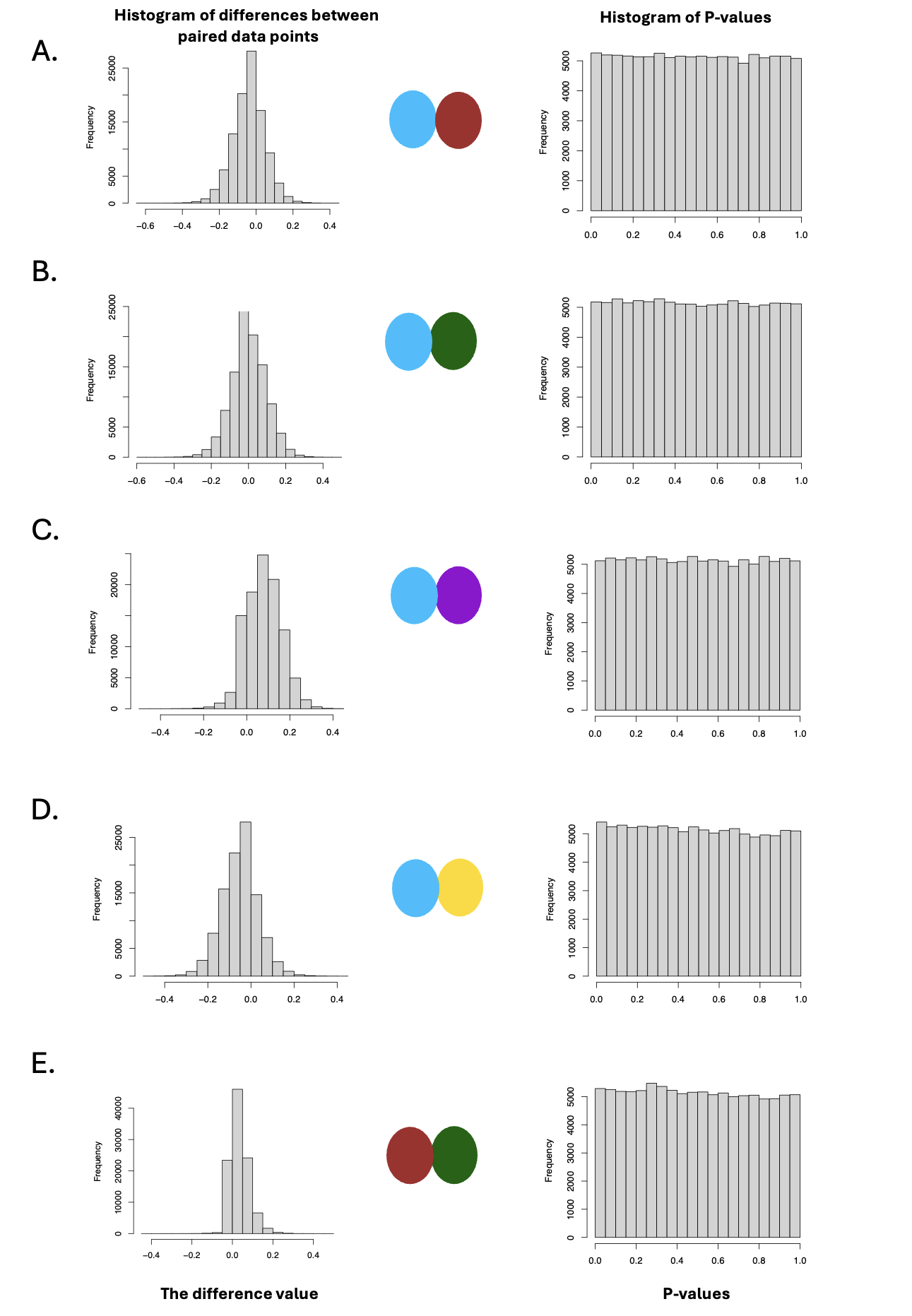

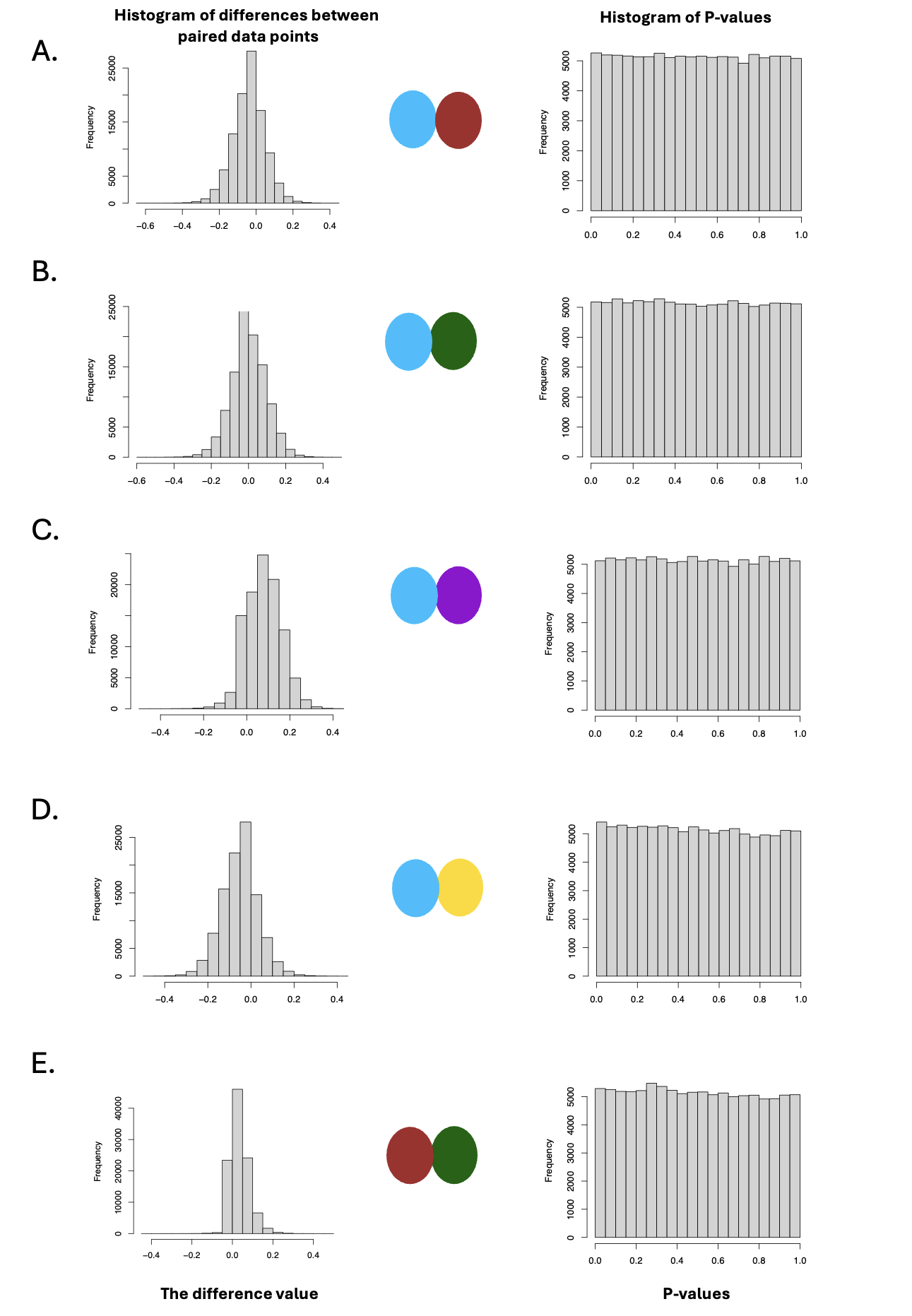

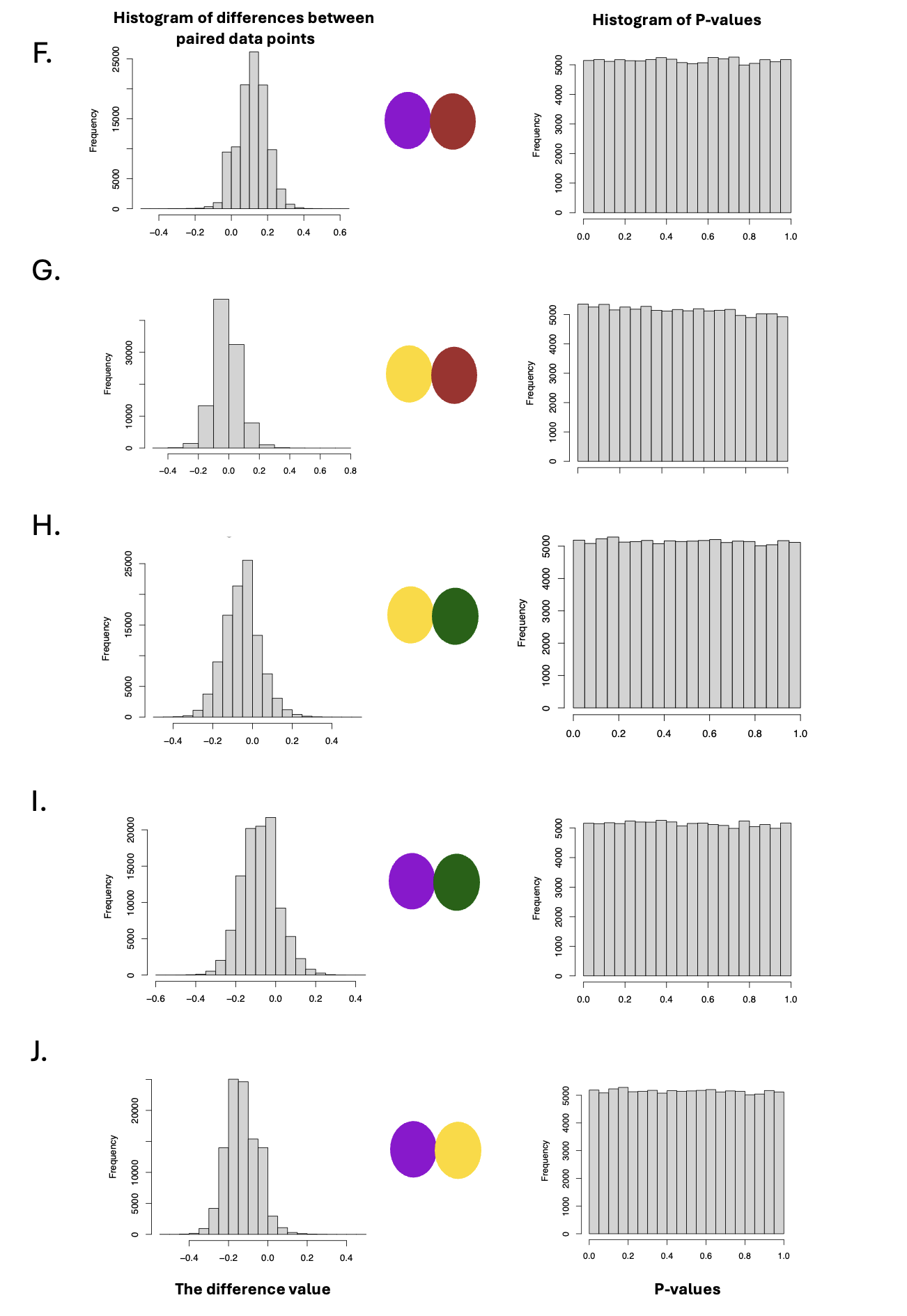


South western Cape

Vs

Great Karoo

South western Cape

Vs

South coastal

South western Cape

Vs

Northern South Africa

Great Karoo

Vs

South coastal

Great Karoo

Vs

Northern South Africa

South coastal

Vs

Northern South Africa

**Figure S9. Histograms of multiple testing results for nucleotide diversity differences between pairs of populations.** Left - histograms of differences between paired data points of nucleotide diversity from two groups. Right - histograms of P-values from multiple tests, showing a homogeneous distribution, indicating no systematic bias in statistical significance across comparisons.

**Figure S10. Population structure of *X. laevis* in South Africa for the L and S sub-genomes.** Results on the left are generated using the SNP dataset of the L- sub-genome, and on the right generated using the SNP dataset of the S- sub-genome. For each dataset, we constructed an unrooted maximum likelihood tree with RAxML and we inferred ancestry proportions with STRUCTURE. Colour code: Blue = South western Cape, Yellow = Northern South Africa, Green = Great Karoo and South coastal, and Purple = Nieuwoudtville.


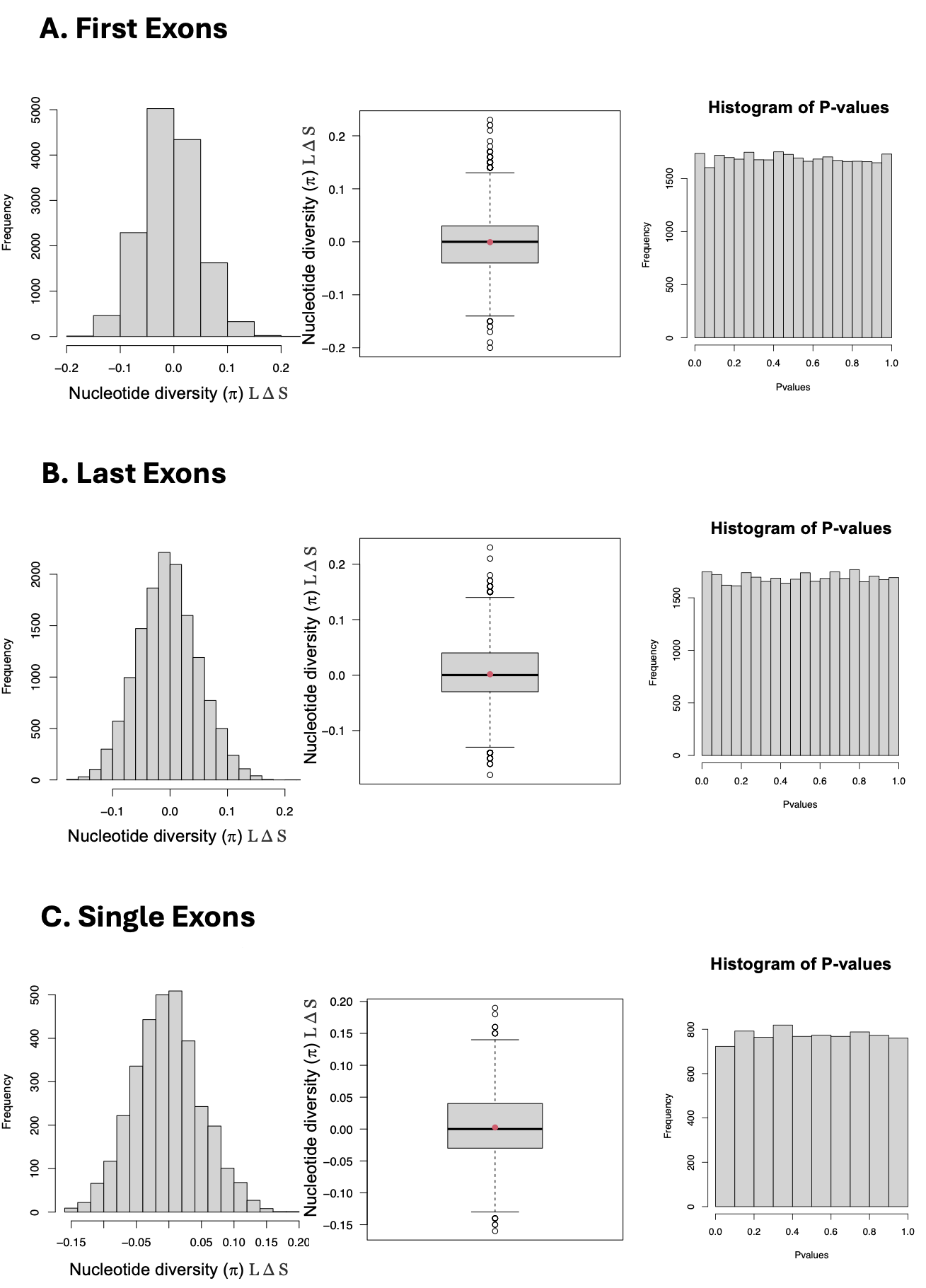


**Figure S11. Multiple testing of nucleotide diversity differences between the L and S sub-genomes for A) the first exons of genes, B) the last exons, and C) single exon genes.** The histograms on the left show the distribution of differences between paired data points of nucleotide diversity from the L and S sub-genomes. The box plots show the nucleotide diversity differences between paired data points from the L and S sub-genomes. The mean is indicated by a red point. The histograms on the right shows the P-values from multiple tests, showing a homogeneous distribution, indicating no systematic bias in statistical significance across comparisons.


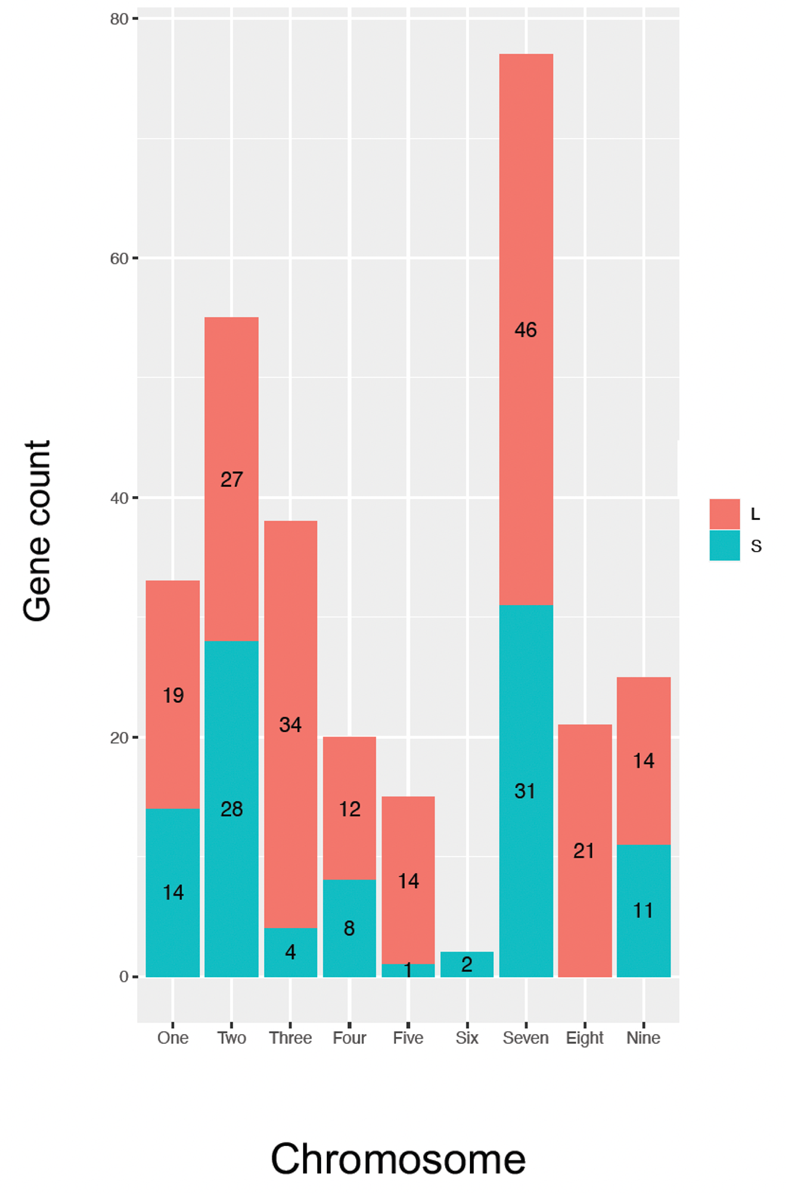


**Figure S12. Chromosomal distribution of genes linked (<10Kb) with highly differentiated SNPs (Fst > 0.15) between the high and low contrasts within the northern South Africa population***.* Pink bars = L sub-genome, and green bars = S sub-genome.

**
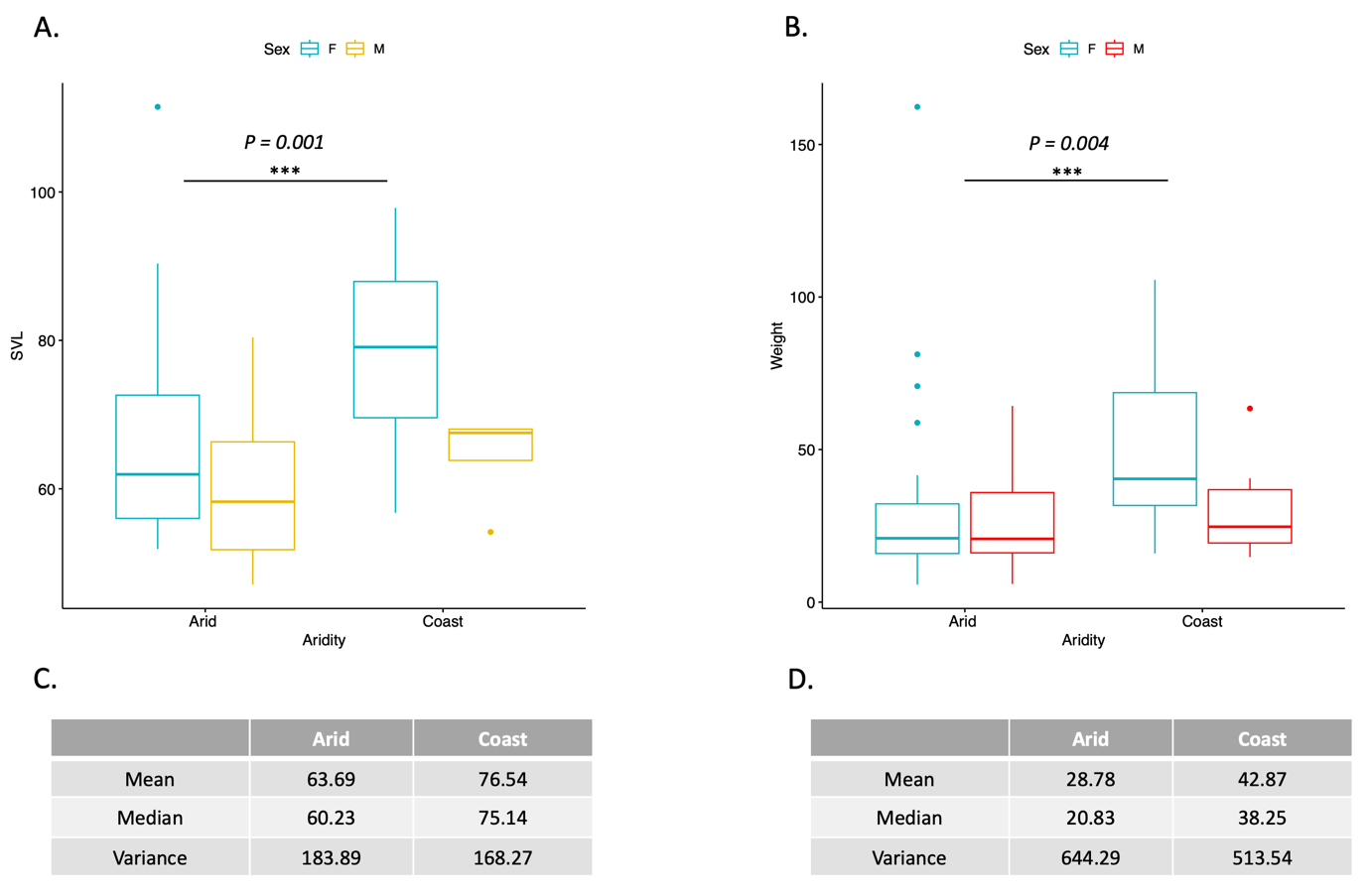
**

# Figure S13. Differences in the Snout to Ventral Length (SVL; cm) and mass (g) between samples collected from arid sites (Great Karoo population) and samples collected from coastal sites (South coastal population). Analysis conducted using morphometrics of 96 individuals, 48 each for the arid (21 males and 27 females) and coastal populations (13 males and 35 females). A) SVL with Wilcoxon signed-rank test results; B) mass; C) summary statistics for SVL D) summary statistics for mass.
